# Hyperinsulinemia drives glomerular podocyte injury and albuminuria *via* a self-perpetuating GSK3β–IRS1 insulin desensitization circuit

**DOI:** 10.1016/j.metabol.2026.156571

**Published:** 2026-02-25

**Authors:** Mengxuan Chen, Yudong Fang, Mingzhuo Zhang, Yan Ge, Shoaib Junejo, Rujun Gong

**Affiliations:** Department of Medicine, University of Toledo College of Medicine, Toledo, OH, USA

**Keywords:** insulin resistance, metabolic dysfunction–associated kidney, disease, insulin pathway, podocytopathy

## Abstract

Epidemiological evidence indicates that hyperinsulinemia is an independent risk factor for albuminuria and renal impairment, yet its molecular basis remains unclear. In prediabetic *db/db* mice, hyperinsulinemia coincided with albuminuria, podocyte injury, and impaired glomerular insulin signaling, characterized by insulin receptor exhaustion and hypoactivity of the IRS1/PI3K/Akt insulin signaling. This led to diminished inhibitory phosphorylation of GSK3β^S9^ in podocytes, denoting GSK3β hyperactivity. Notably, GSK3β co-localized and physically interacted with IRS1 in glomerular podocytes, phosphorylating IRS1^S332^ as a direct substrate. In cultured podocytes, prolonged high insulin exposure induced insulin receptor depletion, GSK3β hyperactivity, and increased inhibitory phosphorylation of IRS1^S332^, forming a self-perpetuating cycle of insulin desensitization and podocyte injury. GSK3β appears to play a key role, as ectopic expression of a constitutively active GSK3β mutant GSK3β^S9A^ enhanced inhibitory phosphorylation of IRS1^S332^, desensitized insulin signaling, and exacerbated podocyte injury. In contrast, forced expression of a kinase-dead mutant of GSK3β or inhibition of GSK3β with a selective small-molecule inhibitor tideglusib abrogated inhibitory phosphorylation of IRS1^S332^, restored insulin sensitivity and protected podocytes. *In vivo*, *GSK3β^S9A^* knock-in mice exhibited impaired insulin signaling and podocyte injury with albuminuria. Conversely, tideglusib treatment in prediabetic *db/db* mice attenuated podocyte injury and insulin resistance, thereby improving albuminuria. Collectively, hyperinsulinemia directly elicits albuminuria and renal impairment *via* a cascade of molecular events involving insulin receptor exhaustion, reduced insulin signaling, and GSK3β hyperactivity, which promotes IRS1 inhibition and thereby forms a self-amplifying GSK3β-IRS1 circuit of insulin desensitization and podocyte injury. Targeting GSK3β could disrupt this pathogenic loop and mitigate hyperinsulinemia-induced renal injury.

## 1. Introduction

Insulin, a vital anabolic hormone secreted by pancreatic islet β-cells, plays a central role in maintaining glucose homeostasis by promoting glucose uptake from the bloodstream into peripheral tissues, where it is utilized for energy production or stored *via* glycogen biogenesis for future use [1]. The insulin signaling cascade is initiated by insulin binding to its cognate insulin receptor, which activates a downstream signaling network involving insulin receptor substrates (IRS) [2], followed by key pathways such as the mitogen-activated protein kinase [3], phosphoinositide 3-kinase (PI3K) [4], and Cbl-associated protein/Cbl [5] pathways. These cascades operate in classic insulin-sensitive tissues, including hepatocytes [6], adipocytes [7] and skeletal muscle cells [1].

Disruption of insulin signaling, whether due to insulin deficiency or resistance, plays a pivotal role in the pathogenesis of metabolic disorders [8], most notably diabetes mellitus [9]. Even in the occult subclinical stages of insulin resistance, attenuated insulin signaling impairs glucose uptake and predisposes to hyperglycemia, triggering compensatory hypersecretion of insulin from pancreatic β-cells in an attempt to maintain normoglycemia [10]. This adaptive response leads to hyperinsulinemia [11]. Hyperinsulinemia and insulin resistance are intimately interrelated metabolic derangements that reciprocally reinforce each another [12]. Over time, this maladaptive cycle contributes to the development and progression of metabolic dysfunction, type 2 diabetes [13], obesity [14], and their associated complications [15,16].

Although traditionally not considered a classic insulin-sensitive effector organ, the kidney is increasingly recognized as an important target of insulin action. Insulin receptors are expressed in multiple renal cell types, including glomerular podocytes [17]. Insulin signaling is essential for maintaining renal homeostasis, as demonstrated by studies showing that podocyte-specific deletion of the insulin receptor in mice results in proteinuria and glomerulosclerosis [18]. Moreover, diabetic kidney disease (DKD), characterized by proteinuria and podocyte injury, represents one of the most common complications of type 2 diabetes involving insulin resistance and hyperinsulinemia [19].

Emerging clinical and epidemiological evidence indicates that hyperinsulinemia, even in the absence of hyperglycemia or diabetes, is an independent risk factor for renal impairment [20]. In prediabetic or non-diabetic conditions such as obesity [21], insulinoma [22], or even in otherwise healthy individuals [23], albuminuria—a hallmark of renal injury—is frequently preceded by or associated with elevated insulin levels. Despite these observational findings, the precise molecular mechanisms underlying the link between hyperinsulinemia and renal injury, particularly albuminuria, remain incompletely understood. Of note, albumin excretion is primarily regulated by the glomerular filtration barrier [24], where podocytes function as critical structural and regulatory components that govern glomerular permselectivity to plasma proteins [25]. Podocyte injury and dysfunction are well-established pathogenic drivers of proteinuric glomerulopathies, including glomerulonephritis [26] and DKD [25]. In this study, we sought to determine whether, and by what mechanisms, hyperinsulinemia directly induces podocyte injury. To this end, we employed prediabetic *db/db* mice as a model of hyperinsulinemia and further validated our findings in cultured podocytes exposed to high insulin.

## 2. Methods

A detailed methods section is provided in the Supplemental Methods.

### 2.1. Animal experiment

All animal experiments and procedures were approved by the University of Toledo Institutional Animal Care and Use Committee and conformed to the regulations of the US Department of Agriculture and the National Institutes of Health Guide for the Care and Use of Laboratory Animals. BKS.Cg-*Dock7*^*m* +*/*+^ *Lepr^db^*/J (*db/m*) mice were purchased from the Jackson Laboratory (#000642, Bar Harbor, ME, USA) and bred to obtain *db/db* mice or control *db/m* mice as described previously [27]. Male mice were given food and water *ad libitum* and were euthanized and examined at 4, 6, 10 weeks of age. In a separate experiment, 4-week-old male *db/db* mice were randomized to receive intraperitoneal injections of tideglusib (TDG, 5 mg/kg; Selleckchem, Houston, TX, USA) on alternate days for 2 weeks. An equal volume of vehicle (corn oil) served as control injections for age-matched male *db/m* and *db/db* mice. Mice were euthanized at 6 weeks of age.

Constitutively active glycogen synthase kinase 3β knock-in (*GSK3β^S9A^ KI*) mice had been originally generated by Dr. Dario Alessi (University of Dundee, UK) and were transferred from Dr. Junichi Sadoshima from Rutgers New Jersey Medical School. Standard breeding protocols were used to breed male heterozygous KI (*GSK3β*^*S9A/*+^) mice and wild-type *(WT)* littermates for the subsequent experiments. Genotyping was carried out by PCR amplification of genomic DNA isolated from ear punched tissues with the following primer pairs: forward: 5’-TCA CTG GTC TAG GGG TGG TGG AAG-3′ and reverse: 5′-GGA GTC AGT GAC AAC ACT TAA CTT-3′, which yield 352-bp and 233-bp amplicons, respectively, for the *KI* and *WT* alleles. Male mice were given food and water *ad libitum* and were euthanized at 8 weeks of age.

### 2.2. Cell culture and treatments

Conditionally immortalized mouse podocytes were cultured in RPMI 1640 medium supplemented with 10% FBS as previously described [28]. After differentiation under nonpermissive conditions, podocytes were maintained in either normal medium or medium containing 100 nM insulin and treated with or without TDG (5 μM) for the indicated times.

### 2.3. Transient transfection and RNA interference (RNAi)

The eukaryotic expression vectors encoding hemagglutinin (HA)- tagged constitutively active (S9A) or dominant-negative kinase-dead (KD) mutants of GSK3β were kindly provided by Dr. Johnson (Birmingham, AL, USA). The murine insulin receptor β-specific siRNA (siIRβ) and the scrambled control siRNA were obtained from Santa Cruz Biotechnology. Podocyte transient transfection was performed using Lipofectamine 3000 or Lipofectamine RNAiMAX (Thermo Fisher) as described before [29]. After transfection, cells were transferred to nonpermissive conditions and maintained in normal growth medium for 48h before assessing transfection or gene-silencing efficiency by immunoblot analysis or immunofluorescence staining. Transfected cells were cultured in normal medium or in medium containing high-dose insulin (100 nM) [30] for the indicated times.

### 2.4. Statistical analysis

All data are expressed as mean ± SD. For immunoblot analysis, band intensities were quantified by densitometric scanning and analyzed using ImageJ software (version 1.53 National Institutes of Health, Bethesda, MD, USA). Normality was assessed using the Shapiro-Wilk test. Statistical analyses were performed with GraphPad Prism 8.0 (GraphPad Software, La Jolla, CA, USA). Comparisons among multiple groups were conducted using one-way ANOVA followed by Tukey's *post hoc* test, and comparisons between two groups were performed using 2-tailed unpaired Studenťs *t-*tests. Linear regression analysis was applied to test possible correlations between two parameters. *P <* 0.05 was considered statistically significant.

## 3. Results

### 3.1. Hyperinsulinemia-associated podocyte dysfunction and albuminuria occur prior to overt hyperglycemia in db/db mice

As a widely used model of type 2 diabetes, *db/db* mice with a defect in the leptin receptor are characterized by hyperinsulinemia [31]. To investigate whether hyperinsulinemia precedes the onset of hyperglycemia, *db/db* mice were examined at early developmental stages, specifically at 4, 6, and 10 weeks of age, representing the prediabetic and early diabetic phases. As shown in Fig. 1A, 4-week-old *db/db* mice exhibited normoglycemia, comparable to control *db/m* mice. By 6 weeks of age, blood glucose levels were negligibly altered, consistent with previous reports [32]. In contrast, at 10 weeks, *db/db* mice developed marked hyperglycemia, with average glucose levels exceeding 300 mg/dL, a level commonly adopted as a diagnostic criterion for established diabetes in murine models [33]. This diabetic state observed at 10 weeks was accompanied by pronounced hyperinsulinemia, as demonstrated by immunoblot analysis of serum samples for insulin (Fig. 1B), and was further associated with significant albuminuria, as quantified by urinary albumin-to-creatinine ratios (uACR) (Fig. 1C). Notably, serum insulin levels in *db/db* mice were elevated as early as 4 weeks of age despite normoglycemia, and became significantly higher by 6 weeks, when albuminuria was already evident (Fig. 1A-D), exhibiting a strong positive correlation with serum insulin levels (Fig. 1E). In parallel, immunoblot analysis of isolated glomeruli revealed a marked reduction in the expression of podocin, a key slit diaphragm protein critical for podocyte homeostasis, in 6-week-old *db/db* mice compared to 4-week-old counterparts. Conversely, the expression of early growth response 1 (EGR1), a marker of podocyte stress or injury, was significantly upregulated (Fig. 1F-H). Interestingly, podocyte numbers were not affected at this stage as indicated by negligible changes in Wilms tumor 1 (WT1) expression (Fig. 1F-H). Furthermore, at 6 weeks, glomerular podocin expression inversely correlated with serum insulin levels (Fig. 1I), suggesting a potential link between hyperinsulinemia and podocyte dysfunction. Immunoblotting results were corroborated by fluorescence immunohistochemistry (Fig. 1H), which demonstrated visibly reduced glomerular podocin expression in 6-week-old prediabetic *db/db* mice.

### 3.2. Glomerular insulin receptor exhaustion and insulin signaling desensitization are associated with hyperinsulinemia in db/db mice at the prediabetic stage

Hyperinsulinemia is intimately interrelated with insulin resistance. Here, shown by immunoblot analysis of isolated glomeruli, glomerular expression of IRβ in the hyperinsulinemic 6-week-old prediabetic *db/db* mice, as opposed to that in control *db/m* mice, was significantly reduced (Fig. 2A), indicative of glomerular insulin receptor exhaustion, suggesting that insulin resistance is concomitant with prediabetic hyperinsulinemia. To test the insulin signaling activity, glomeruli were isolated from 6-week-old *db/db* mice or control *db/m* mice and treated *ex vivo* (Fig. 2B) with insulin. PI3K/Akt is a major signaling pathway activated by insulin that could be detected by Akt phosphorylation. Shown in Fig. 2C-D, insulin-induced Akt phosphorylation was considerably blunted in glomeruli derived from the prediabetic *db/db* mice. This was associated with a suppression in insulin-induced glucose uptake, as a functional readout of insulin action (Fig. 2E).

### 3.3. Glomerular GSK3β overexpression and hyperactivity is associated with hyperinsulinemia and albuminuria in prediabetic db/db mice

As a downstream transducer of insulin-activated PI3K/Akt pathway, GSK3β has been recognized for its critical role in regulating glycogen synthesis and recently been involved in glomerular disease and podocyte injury [34]. However, whether and how it plays a role in hyperinsulinemia-associated renal impairment is unknown. To address this issue, glomerular expression of GSK3β and inhibitorily phosphorylated GSK3β at serine 9 was examined by immunoblot analysis, which showed reduced expression of p-GSK3β^S9^ but increased expression of GSK3β, denoting GSK3β overexpression and hyperactivity, as indicated by a significantly suppressed ratio of p-GSK3β^S9^/GSK3β (Fig. 3A). Consistent with this finding, increased glomerular GSK3β expression has been evidenced by glomerular transcriptomic analysis in KK*-A^y^* mice with type 2 diabetes (Supplemental Fig. 1), as well as in kidney biopsies from patients with diabetic nephropathy (DN) (Supplemental Fig. 2A). In prediabetic *db/db* mice, glomerular GSK3β hyperactivity occurred predominantly in podocytes, as evidenced by dual-color fluorescent immunohistochemistry for podocin and p-GSK3β^S9^, which demonstrated that glomerular expression of p-GSK3β^S9^ was abundant in podocin-positive podocytes in *db/m* mice but was markedly diminished in prediabetic *db/db* mice (Fig. 3B-C). This result serendipitously aligns with the findings from *post hoc* analyses of publicly available glomerular RNA-seq transcriptomic datasets across species. Specifically, analysis of single-cell RNA sequencing (scRNA-seq) data from the Chung study [35] demonstrated that podocyte expression of GSK3β was markedly higher in *ob/ob* (BTBR.Cg*-Lep^ob^/WiscJ)* mice, which are known to sustain insulin resistance due to leptin deficiency, than in control *ob/*+ mice (Fig. 3D-E). Besides, single-nucleus RNA sequencing (snRNA-seq) transcriptomic profiling of human kidney specimens from the Wilson study [36] revealed podocyte-specific overexpression of GSK3β in patients with early DN compared with non-diabetic control subjects, denoting podocyte GSK3β hyperactivity at early stage of the disease (Supplemental Fig. 2B-D). In addition, linear regression analysis indicated a significant negative correlation between serum insulin levels and the ratio of glomerular p-GSK3β^S9^/GSK3β (Fig. 3F), as well as between the ratio of glomerular p-GSK3β^S9^/GSK3β and uACR (Fig. 3G). In contrast, a positive correlation was observed between the ratio of glomerular p-GSK3β^S9^/GSK3β and glomerular podocin (Fig. 3H). These findings suggest that a link between hyperinsulinemia and glomerular GSK3β hyperactivity and a link between glomerular GSK3β hyperactivity and albuminuria or podocyte injury exist in prediabetic *db/db* mice.

### 3.4. GSK3β interacts with IRS1 and drives its inhibition in hyperinsulinemia-associated podocyte injury

To further substantiate the role of GSK3β in hyperinsulinemia-induced podocyte injury, genes associated with this pathological process were retrieved from the GeneCards database and integrated into the STRING database to construct a protein–protein interaction network (Supplemental Fig. 3). Consistent with the findings above in prediabetic *db/db* mice, GSK3β was identified as a central hub gene, exhibiting a high protein interaction score. Notably, among other key hub genes, IRS1, a critical proximal mediator of insulin signaling, displayed a high degree of connectivity and a short path length with GSK3β (Fig. 4A). To confirm these findings, a molecular docking analysis was conducted. As shown in Fig. 4B, multiple residue clusters were predicted to form hydrogen bonds at the GSK3β-IRS1 interface, supporting a potential physical association between the two proteins. For instance, a hydrogen bond was identified between Arg111 of GSK3β and Cys209 of IRS1. The docking score for the GSK3β-IRS1 complex was estimated to be − 454, consistent with a stable interaction. To validate this bioinformatics-predicted protein interaction, the subcellular spatial relationship between GSK3β and IRS1 was examined next by dual-color fluorescence immunocytochemistry (Fig. 4C). Confocal microscopy of cultured podocytes revealed a distinct pool of GSK3β co-localizing with IRS1. Frequency scatterplots generated by ImageJ-based co-localization analysis demonstrated a Spearman's correlation coefficient of 0.7749 between GSK3β and IRS1 signals, indicative of significant co-localization (Fig. 4C).

To verify a potential physical interaction between GSK3β and IRS1, co-immunoprecipitation assays were conducted and demonstrated that GSK3β co-precipitated with IRS1 in podocyte lysates, supporting that GSK3β physically interacts with IRS1 in podocytes. Notably, this interaction was reinforced following exposure to high insulin, as evidenced by increased IRS1 co-precipitation with GSK3β (Fig. 4D). In light of previous studies identifying IRS1 as a potential substrate of GSK3β kinase activity [37], these findings imply that insulin stimulation may augment GSK3β-mediated phosphorylation of IRS1. Indeed, in an *in vitro* model of hyperinsulinemia-induced podocyte injury (Fig. 4E-H), exposure to high insulin for varying durations (30 min, 8 h, 24 h, and 10 days) led to progressive desensitization of insulin signaling, characterized by IRβ depletion and sustained increase in IRS1 phosphorylation at Ser332, a modification known to impair IRS1 signaling function [37]. This inhibition of IRS1 occurred concomitantly with increased GSK3β activity, as evidenced by a gradual decline in the p-GSK3β^S9^/GSK3β ratio, despite an initial transient rise. Consistent with these *in vitro* findings, immunoblot analysis of glomeruli isolated from prediabetic *db/db* mice demonstrated elevated p-IRS1^S332^ levels accompanied by reduced expression of total IRS1 (Fig. 4I). Furthermore, linear regression analysis revealed a significant inverse correlation between the glomerular p-GSK3β^S9^/GSK3β ratio and the p-IRS1^S332^/IRS1 ratio (Fig. 4J), supporting the hypothesis that GSK3β hyperactivity contributes to IRS1 inhibition in the context of hyperinsulinemia-associated glomerular injury.

### 3.5. Podocyte GSK3β hyperactivity also contributes to impaired insulin signaling in streptozotocin (STZ)-induced type 1 DKD

To determine whether GSK3β hyperactivity is also present in type 1 DN and contributes to impaired insulin signaling, a murine model of STZ-elicited type 1 diabetes was examined (Supplemental Fig. 4A). Podocyte injury was evident in mice with STZ-induced DKD, marked by reduced glomerular expression of the podocyte homeostatic marker podocin and increased expression of the injury marker EGR1, based on immunoblot analysis of isolated glomeruli (Supplemental Fig. 4B). In parallel, dual color fluorescent immunohistochemical staining demonstrated diminished staining of inhibitorily phosphorylated GSK3β^S9^ in podocin-positive glomerular podocytes, indicative of GSK3β hyperactivity in podocytes. These morphological findings were further confirmed by immunoblot analysis of isolated glomeruli (Supplemental Fig. 4C–D). This result is concordant with a *post-hoc* analysis of the publicly available glomerular transcriptomic dataset from mice with STZ-induced DKD reported by Cao et al. [38] (Supplemental Fig. 4E), which revealed significantly increased glomerular GSK3β expression in STZ-treated mice compared with controls, further supporting GSK3β hyperactivity in type 1 DN. Moreover, in our mice with STZ-induced type 1 DKD, a positive correlation was observed between the ratio of glomerular p-GSK3β^S9^/GSK3β and glomerular expression of podocin (Supplemental Fig. 4F), supporting a link between GSK3β hyperactivity and podocyte injury in type 1 DN. Moreover, immunoblot analyses of isolated glomeruli revealed impaired insulin signaling, indicated by reduced Akt phosphorylation, increased inhibitory phosphorylation of IRS1 along with an elevated p-IRS1^S332^/IRS1 ratio, despite negligible changes in IRβ expression (Supplemental Fig. 4G-H). Furthermore, linear regression analysis revealed an inverse correlation between glomerular p-GSK3β^S9^/GSK3β and p-IRS1^S332^/IRS1 ratios (Supplemental Fig. 4I), suggesting GSK3β-dependent inhibitory regulation of IRS1 in type 1 DN.

### 3.6. GSK3β controls insulin signaling sensitivity and podocyte injury under hyperinsulinemia conditions

To establish a direct causal relationship between GSK3β signaling and insulin sensitivity, GSK3β activity was modulated in cultured podocytes *via* forced expression of either an empty plasmid vector (EV), or plasmids encoding HA-tagged S9A or KD mutant of GSK3β in an *in vitro* model of hyperinsulinemia-induced podocyte injury. Upon prolonged exposure to high insulin, podocyte injury, characterized by decreased expression of the podocyte marker synaptopodin and increased expression of EGR1, was exacerbated by S9A expression but attenuated by KD expression, as confirmed by both immunofluorescence and immunoblot analyses (Fig. 5A-C). Aberrant podocyte motility, a known contributor to podocyte dysfunction, was also assessed using a conventional cell migration assay. S9A expression enhanced podocyte motility, as evidenced by increased gap closure between the invading fronts of podocyte sheets, whereas KD expression suppressed this response (Fig. 5D-E). These injury phenotypes were associated with corresponding alterations in insulin action. Functionally, insulin-stimulated glucose uptake, a direct measure of insulin action, was further impaired in S9A-expressing podocytes but improved in KD-expressing cells (Fig. 5F). In addition, despite comparable levels of IRβ expression across groups, phosphorylation of IRS1 at Ser332 and the p-IRS1^S332^/IRS1 ratios were significantly elevated in S9A-expressing cells but reduced in KD-expressing cells (Fig. 5G-H). These changes coincided with decreased Akt phosphorylation and lower p-Akt/Akt ratios in S9A-expressing cells, and conversely, with enhanced Akt phosphorylation and higher p-Akt/Akt ratios in KD-expressing cells, respectively indicative of aggravated and ameliorated insulin resistance (Fig. 5G-H).

To assess whether insulin receptor exhaustion contributes to hyperinsulinemia-induced insulin resistance and podocyte injury through a GSK3β-dependent mechanism, cultured podocytes were co-transfected with either siIRβ or control siRNA (siCtrl), coupled with plasmid vectors encoding either an EV or KD mutant of GSK3β. Following transfection, cells were maintained under hyperinsulinemic conditions. As shown in Fig. 5I-J, IRβ silencing as opposed to control-RNAi treatment substantially diminished IRβ expression. This resulted in an impaired insulin signaling, marked by reduced phosphorylation of Akt. This coincided with elevated phosphorylation of IRS1^S332^, denoting IRS1 suppression. These signaling alterations were accompanied by clear signs of podocyte injury, including reduced synaptopodin expression and increased EGR1 levels. In contrast, in podocytes expressing the KD mutant of GSK3β, despite a comparable degree of IRβ knockdown, insulin signaling impairment was significantly averted, as evidenced by blunted IRS1^S332^ phosphorylation and preserved levels of Akt phosphorylation. Accordingly, podocyte injury was attenuated, as evidenced by preserved synaptopodin expression and diminished EGR1 induction (Fig. 5I-J). Collectively, these data suggest the existence of a self-reinforcing pathological feedforward loop, wherein prolonged hyperinsulinemia exacerbates insulin resistance through GSK3β-mediated suppression of IRS1. This, in turn, further desensitizes the PI3K/Akt arm of the insulin signaling pathway and promotes sustained GSK3β hyperactivity (Fig. 5K).

### 3.7. Constitutively active GSK3β^S9A^ KI mice phenotypically recapitulate hyperinsulinemia-associated glomerular injury in the absence of elevated insulin

To determine whether GSK3β hyperactivity is sufficient to cause hyperinsulinemia-associated glomerular injury, *GSK3β^S9A^ KI* mice were examined alongside *WT* littermates (Fig. 6A). Immunoblot analysis of isolated glomeruli confirmed a marked reduction in the inhibitory phosphorylation of GSK3β at Ser9 in *GSK3β^S9A^ KI* mice at 8 weeks of age (Fig. 6A), indicating pronounced GSK3β hyperactivity. This was associated with clear evidence of podocyte injury, as demonstrated by immunoblotting and immunohistochemistry, including significant loss of podocin expression and elevated EGR1 levels, despite negligible changes in podocyte number, as assessed by WT1 staining (Fig. 6B-C).

Consequently, *GSK3β^S9A^ KI* mice excreted significantly higher levels of albumin in urine compared to *WT* controls, as assessed by uACR and urine protein electrophoresis (Fig. 6D-E), while serum albumin levels remained comparable between the two groups (Fig. 6F). The albuminuria observed in *GSK3β^S9A^ KI* mice was unlikely to result from hemodynamic alteration, as both systolic and diastolic blood pressures, as well as cardiac functions like ejection fraction were similar to those of *WT* mice, based on tail-cuff measurements and echocardiogram (Fig. 6G-H). Importantly, *GSK3β^S9A^ KI* mice displayed normal blood glucose and insulin levels (Fig. 6I-J), and no differences in glomerular expression of IRβ were observed (Fig. 6K). However, phosphorylation of IRS1^S332^ was significantly elevated in *GSK3β^S9A^ KI* mice, coinciding with impaired insulin signaling in glomeruli, as evidenced by reduced Akt phosphorylation and lower p-Akt/Akt ratios (Fig. 6K). Of note, GSK3β knock-in barely affected glomerular β-catenin signaling, a canonical downstream pathway of GSK3β, as shown by comparable levels of nuclear β-catenin between *GSK3β^S9A^ KI* and *WT* mice by immunoblot analysis (Supplemental Fig. 5).

Consistent with the *in vivo* findings, *in vitro* in immortalized podocytes transfected with plasmids encoding GSK3β^S9A^ (Fig. 6L-M), Akt phosphorylation was blunted, while the p-IRS1^S332^/IRS1 ratio was significantly elevated. Furthermore, forced expression of S9A elicited podocyte injury, evidenced by increased expression of EGR1 and decreased expression of synaptopodin on immunoblot analysis (Fig. 6L-M). These findings suggest that hyperactive GSK3β recapitulates the hyperinsulinemia-induced podocyte injury and insulin desensitization both *in vivo* and *in vitro*, without perturbing systemic glucose or insulin homeostasis (Fig. 6N).

### 3.8. Pharmacological inhibition of GSK3β by tideglusib protects against hyperinsulinemia-associated podocyte injury and improves insulin sensitivity

Numerous highly selective small-molecule inhibitors have been developed with high specificity for GSK3β. Among them, TDG is the only GSK3β inhibitor that has completed phase II clinical trials for conditions, such as Alzheimer's disease, congenital myotonic dystrophy type 1, progressive supranuclear palsy, and amyotrophic lateral sclerosis, in which TDG has been shown to be safe and well tolerated, with minimal side effects [39–41]. To evaluate the pharmacological effect of TDG on hyperinsulinemia-induced podocyte injury, cultured podocytes were maintained in normal medium or exposed to high insulin levels, followed by treatment with or without TDG. As shown in Fig. 7A, TDG treatment partially restored the inhibitory phosphorylation of GSK3β in podocytes insulted with high insulin, indicating successful abrogation of GSK3β hyperactivity. This was associated with attenuated podocyte injury, as evidenced by preserved synaptopodin expression and reduced EGR1 induction, confirmed by both immunofluorescence and immunoblot analyses (Fig. 7A–B).

Moreover, TDG treatment delayed closure of the gap between the advancing fronts of podocyte sheets in a cellular migration assay (Fig. 7C–D), suggesting correction of the hypermotility induced by prolonged insulin exposure. TDG also improved insulin sensitivity in injured podocytes, as demonstrated by enhanced glucose uptake compared to vehicle-treated controls (Fig. 7E). Mechanistically, TDG increased Akt phosphorylation and decreased phosphorylation of IRS1^S332^, as well as the p-IRS1^S332^/IRS1 ratio, without significantly affecting IRβ expression as demonstrated by immunoblot analysis (Fig. 7F).

To determine the effect of TDG *in vivo*, prediabetic *db/db* mice were treated with TDG or vehicle for 2 weeks (Fig. 8A). TDG treatment significantly reduced hyperinsulinemia-associated albuminuria, as assessed by uACR (Fig. 8B) and urine protein electrophoresis (Fig. 8C). This was concomitant with a podocyte-sparing effect, as evidenced by preserved glomerular podocin expression and diminished EGR1 induction (Fig. 8D–E). Consistent with the *in vitro* findings, the protective effect of TDG was associated with an enhanced inhibition of GSK3β in podocytes *in vivo*, as evidenced by increased expression of p-GSK3β^S9^ in podocin-positive glomerular podocytes (Fig. 8D) and corroborated by immunoblot analysis (Fig. 8E-F). Moreover, TDG restored insulin signaling in glomeruli, as reflected by increased Akt phosphorylation and decreased phosphorylation of IRS1^S332^, as well as the p-IRS1^S332^/IRS1 ratios, without significantly affecting IRβ expression (Fig. 8G-H).

## 4. Discussion

Hyperinsulinemia is a common clinical finding that coexists with a constellation of metabolic disorders, including obesity [42], hypertension [16], insulin resistance [12], type 2 diabetes mellitus [43], and cardiovascular diseases [44]. Hyperinsulinemia is also an independent risk factor for renal impairment particularly marked by albuminuria [45], yet its underlying pathomechanism remains unclear. In this study, we delineate a sequence of key signaling events in glomerular podocytes in response to sustained hyperinsulinemia. Specifically, hyperinsulinemia triggers GSK3β hyperactivity in podocytes, which in turn promotes a self-reinforcing feedforward cycle of insulin signaling impairment *via* GSK3β-IRS1 axis, driving podocyte injury and albuminuria. To our knowledge, this study is the first to demonstrate the deleterious effect of hyperinsulinemia on glomerular podocytes.

A growing body of clinical evidence suggests that hyperinsulinemia disrupt renal homeostasis [45]. In type 2 diabetes, insulin resistance is frequently associated with microalbuminuria [46]. Notably, in a prospective cohort study of normoalbuminuric type 2 diabetic patients, insulin resistance significantly predicted the development of microalbuminuria [46]. Despite confounding by hyperglycemia, hyperinsulinemia plays a central and direct role in renal injury [47,48]. Supporting this, in healthy individuals, exogenous insulin increased renal blood flow and glomerular filtration rate [49]. Moreover, a prospective study in non-diabetic subjects [47] demonstrated that elevated fasting insulin and insulin resistance preceded the onset of albuminuria, implicating hyperinsulinemia as an early driver of renal injury. Consistently, the relationship between insulin sensitivity and cardiovascular disease (RISC) study [50] indicated that reduced insulin sensitivity was continuously associated with an increased risk of albuminuria progression in healthy, non-diabetic, middle-aged adults. The detrimental effect of hyperinsulinemia on the kidney, independent of hyperglycemia, has also been observed in animal models. In prediabetic Otsuka Long-Evans Tokushima fatty (OLETF) rats, renal structural and functional alterations occurred in the setting of hyperinsulinemia without overt hyperglycemia [20]. Similarly, using prediabetic *db/db* mice as a model of isolated hyperinsulinemia, we observed early podocyte injury and albuminuria. To isolate the renal effects of hyperinsulinemia, Monu et al. [51] infused insulin directly into left kidneys in rats, using the contralateral kidney as a control. After six weeks, insulin-infused kidneys developed significant glomerular injury, including glomerular basement membrane thickening, despite normoglycemia, normal systemic insulin levels, and unchanged blood pressure, indicating that localized hyperinsulinemia alone is sufficient to induce renal damage.

In our study, prolonged exposure to high ambient insulin exerted a direct deleterious effect on podocytes. This was associated with desensitization of insulin signaling. This result partially aligns with findings in the OLETF rat model, where hyperinsulinemia-associated renal injury was accompanied by suppression of the IRS1/PI3K/Akt pathway in kidney tissues, and also in cultured renal tubular cells exposed to high insulin [20]. Notably, our findings indicate that, under conditions of attenuated IRS1/PI3K/Akt signaling, hyperactive GSK3β plays an instrumental role in initiating a self-reinforcing feedforward loop *via* direct inhibitory phosphorylation of IRS1, which is a *bona fide* substrate of GSK3β [52]. This feedforward mechanism perpetuates insulin signaling impairment and podocyte dysfunction. Our mechanistic model appears to be operative not only in type 2 DKD but also in type 1 DKD, wherein insulin signaling is initially disrupted by insulin deficiency rather than hyperinsulinemia-induced insulin resistance, as observed in type 2 DKD. This interpretation is line with a previous report by Cheng et al. [53], which demonstrated reduced inhibitory phosphorylation of GSK3β^S9^ in the kidneys of mice with STZ-induced DKD, accompanied by impaired insulin signaling in the kidneys, as evidenced by reduced phosphorylation of PI3K and Akt.

Recent studies have implicated GSK3β as a central convergent point of multiple podocytopathic pathways [34,54,55], including those involved in cytoskeletal remodeling and maintenance of the slit diaphragm [56]. In light of the findings here, GSK3β emerges as a critical mediator in hyperinsulinemia-associated renal impairment, owing to its dual role in both insulin signaling desensitization and the podocytopathic pathways. Indeed, *GSK3β^S9A^ KI* was sufficient to recapitulate hyperinsulinemia-associated renal impairment. Given that high-calorie diets rapidly impair insulin signaling and induce systemic insulin resistance [57], it is plausible that *GSK3β^S9A^ KI* mice, when subjected to a high-calorie diet, would exhibit exacerbated renal injury due to a synergistic effect of GSK3β hyperactivity and high-calorie diet on impairing insulin signaling.

As an actionable therapeutic target, GSK3β hyperactivity in prediabetic *db/db* mice can be averted by TDG, a clinically relevant small-molecule inhibitor of GSK3β with a reported favorable safety profile [40,41]. Consistently, Marcella et al. [58] showed that TDG can enhance insulin sensitivity in experimental Duchenne muscular dystrophy. Nevertheless, our study is limited to preclinical models, and the effects of TDG on hyperinsulinemia-associated kidney injury in humans remain unknown and warrant further clinical and translational investigation. In addition to TDG, lithium has long been established as a standard pharmacological inhibitor of GSK3β in preclinical research [59]. Although lithium is an FDA-approved mood stabilizer primarily indicated for psychiatric disorders, a growing body of experimental and clinical evidence suggests that it can also enhance insulin sensitivity. For example, Rossetti reported that two weeks of lithium treatment completely restored insulin sensitivity in diabetic rats [60]. Similarly, a retrospective chart review found that psychiatric patients receiving lithium exhibited a significant decrease in fasting plasma glucose after one year [61]. Besides, a prospective study of psychotic and manic-depressive patients demonstrated that lithium treatment was associated with significantly reduced plasma insulin levels, consistent with improved insulin signaling [61]. Conversely, transient diabetes has been reported following withdrawal of lithium therapy in psychiatric patients [62], further supporting the insulin-sensitizing effects of lithium. Collectively, these findings provide translational support for our mechanistic model.

In summary, our study demonstrates that hyperinsulinemia alone, in the absence of hyperglycemia, is sufficient to cause renal impairment characterized by podocyte injury and albuminuria. Mechanistically, sustained high insulin leads to insulin receptor exhaustion and inhibition of the IRS1/PI3K/Akt signaling pathway, resulting in GSK3β hyperactivation. GSK3β promotes inhibitory phosphorylation of IRS1, establishing a self-perpetuating feedforward loop that exacerbates insulin resistance and podocyte dysfunction (Fig. 9). Targeting GSK3β could disrupt this pathogenic loop and may offer a promising strategy for preventing renal injury in hyperinsulinemic states.

## Data availability

Renal glomerular transcriptome data from the Ju CKD Glom study [63] were acquired from Nephroseq (www.Nephroseq.org). Glomerular transcriptomic data from Sato study [64] were acquired from the Gene Expression Omnibus (GEO) database under accession number GSE261386. Glomerular transcriptomic data from Cao study [38] were acquired from GEO (GSE168676). Renal scRNA-seq transcriptomic data from the Chung study [35] were acquired from the Single Cell Portal (https://singlecell.broadinstitute.org/single_cell) under the accession number SCP975. Renal snRNA-seq transcriptomic data from the Wilson study [36] were accessed *via* the Kidney Interactive Transcriptomics (K. I.T.) portal (https://humphreyslab.com/SingleCell/). Additional data used in this study were obtained from the GeneCards (https://www.genecards.org) and incorporated into the STRING (https://string-db.org) database for protein–protein interaction analysis. All data generated or analyzed in this study are included in this article. Other source data supporting the findings of this study are available from the corresponding author upon reasonable request.

## Supplementary Material

1

## Figures and Tables

**Fig. 1.**
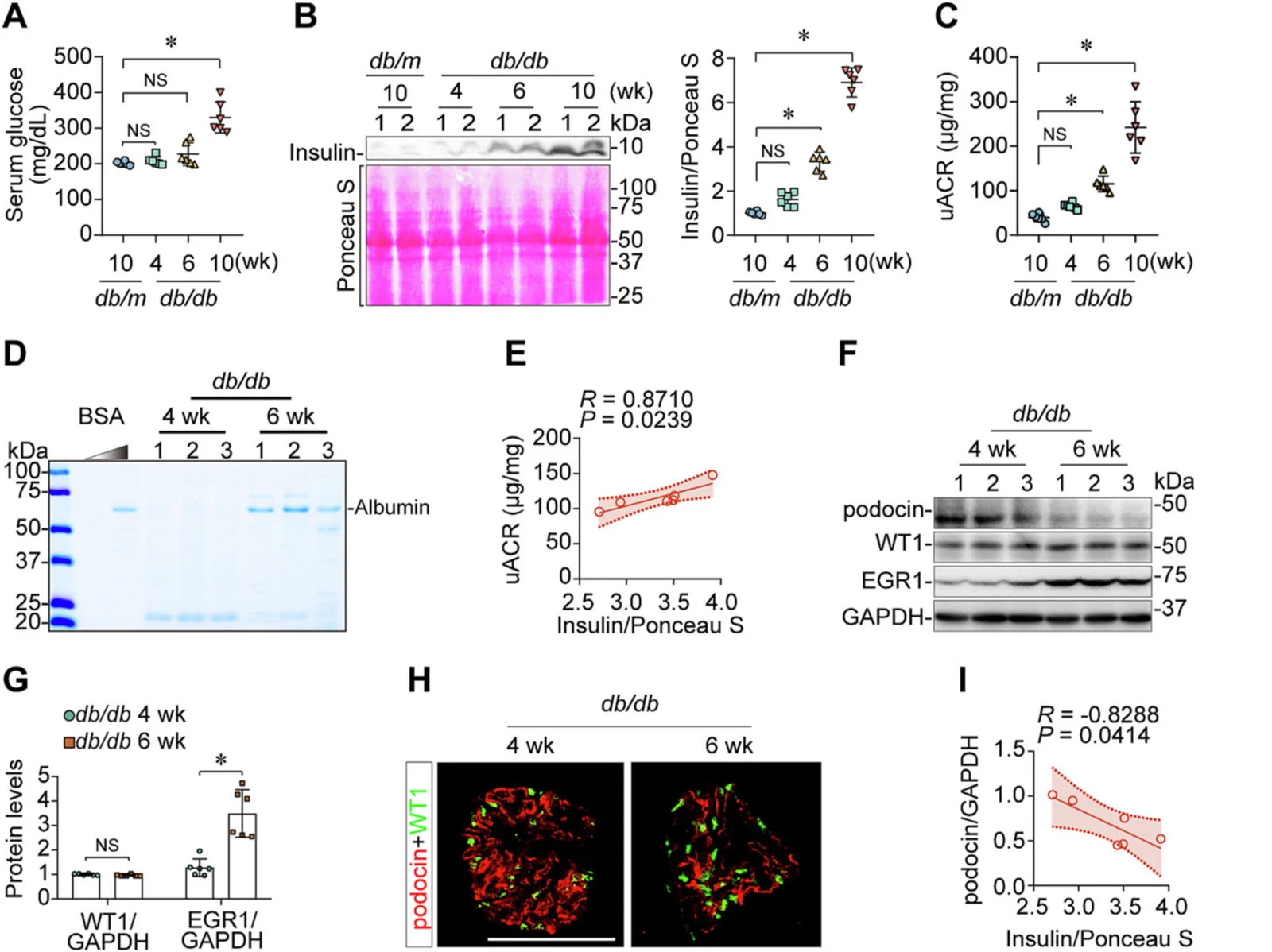
Hyperinsulinemia precedes the onset of hyperglycemia and is associated with podocyte injury and albuminuria in pre-diabetic *db/db* mice. A) Serum glucose levels in *db/db* mice were measured at the indicated ages. Ten-week(wk)-old db/m mice served as the control group. *P < 0.05; NS, non-significance (*n* = 6). (B) Representative immunoblot analysis of serum insulin. Ponceau S staining served as a loading control. Densitometric quantification of insulin expression is shown as relative levels normalized to Ponceau S staining. *P < 0.05; NS, non-significance (*n* = 6). (C) Urinary albumin excretion was assessed by measuring the urinary albumin-to-creatinine ratio (uACR) at the indicated ages in *db/m* and *db/db* mice. *P < 0.05; NS, non-significance (n = 6). (D) Spot urine collected at the indicated ages was subjected to SDS-PAGE followed by Coomassie Brilliant Blue staining. Bovine serum albumin (BSA, 0.5 μg) served as a standard. (E) Linear regression analysis reveals significant correlations between the relative serum levels of insulin in Fig. 1B and uACR in Fig. 1C (n = 6). Spearman's correlation coefficient (*R*) and *P* value are shown. (F) Representative immunoblot analysis of isolated glomeruli for indicated proteins. GAPDH served as a loading control. (G) Densitometric analysis of the expression levels of the indicated proteins, presented as relative levels normalized to GAPDH based on immunoblot analysis. *P < 0.05; NS, non-significance (n = 6). (H) Representative fluorescent micrographs of dual-color fluorescence staining for podocin (red) and Wilms tumor 1 (WT1, green) in frozen murine kidney sections (Scale bar, 50 μm). (I) Linear regression analysis reveals significant correlations between the relative serum levels of insulin in Fig. 1B and relative expression of podocin based on densitometric analysis of immunoblots in Fig. 1F (n = 6). Spearman's correlation coefficient (*R*) and *P* value are shown.

**Fig. 2.**
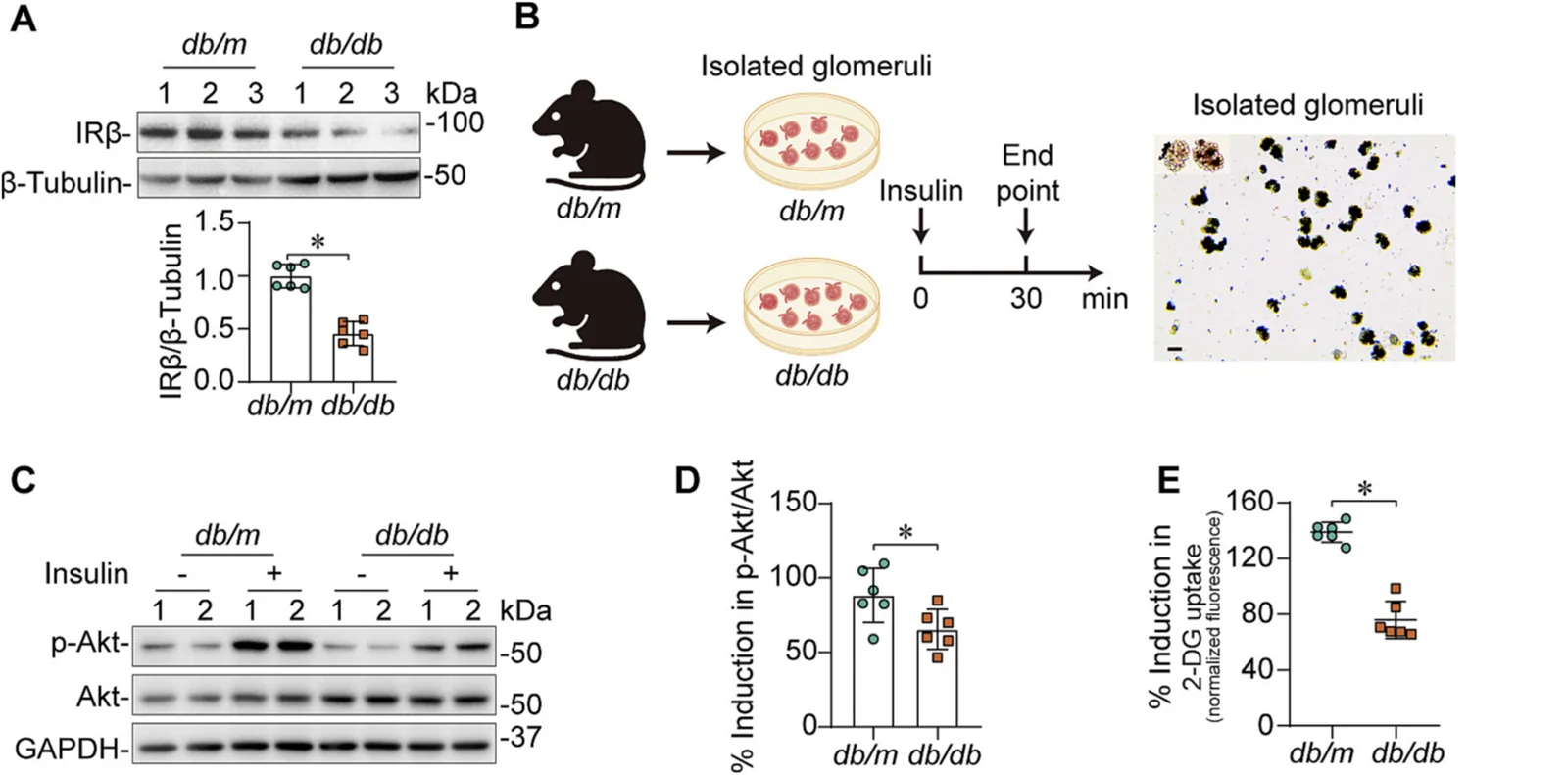
Glomerular insulin receptor exhaustion in pre-diabetic *db/db* mice is associated with insulin signaling desensitization. (A) Immunoblot analysis of isolated glomeruli for insulin receptor (IR) β. β-Tubulin served as a loading control. Densitometric analysis of the relative expression levels of IRβ, presented as relative levels normalized to β-Tubulin based on immunoblot analysis. *P < 0.05 (n = 6). (B) Schematic diagram illustrates *ex vivo* cultures of glomeruli isolated from *db/m* and *db/db* mice at 6 weeks old and treated with medium with or without high insulin (100 nM) for 30 min. Representative micrograph shows cultured glomeruli (Scale bar, 50 μm). (C) Immunoblot analysis of cultured glomeruli for indicated proteins. GAPDH served as a loading control. (D) Percentage induction of in the expression of p-Akt/Akt, relative to the respective vehicle-treated groups. *P < 0.05 (*n* = 6). (E) Percentage induction of 2-deoxyglucose (2-DG) uptake by insulin in *ex vivo* glomeruli, relative to their respective vehicle-treated groups. *P < 0.05 (n = 6).

**Fig. 3.**
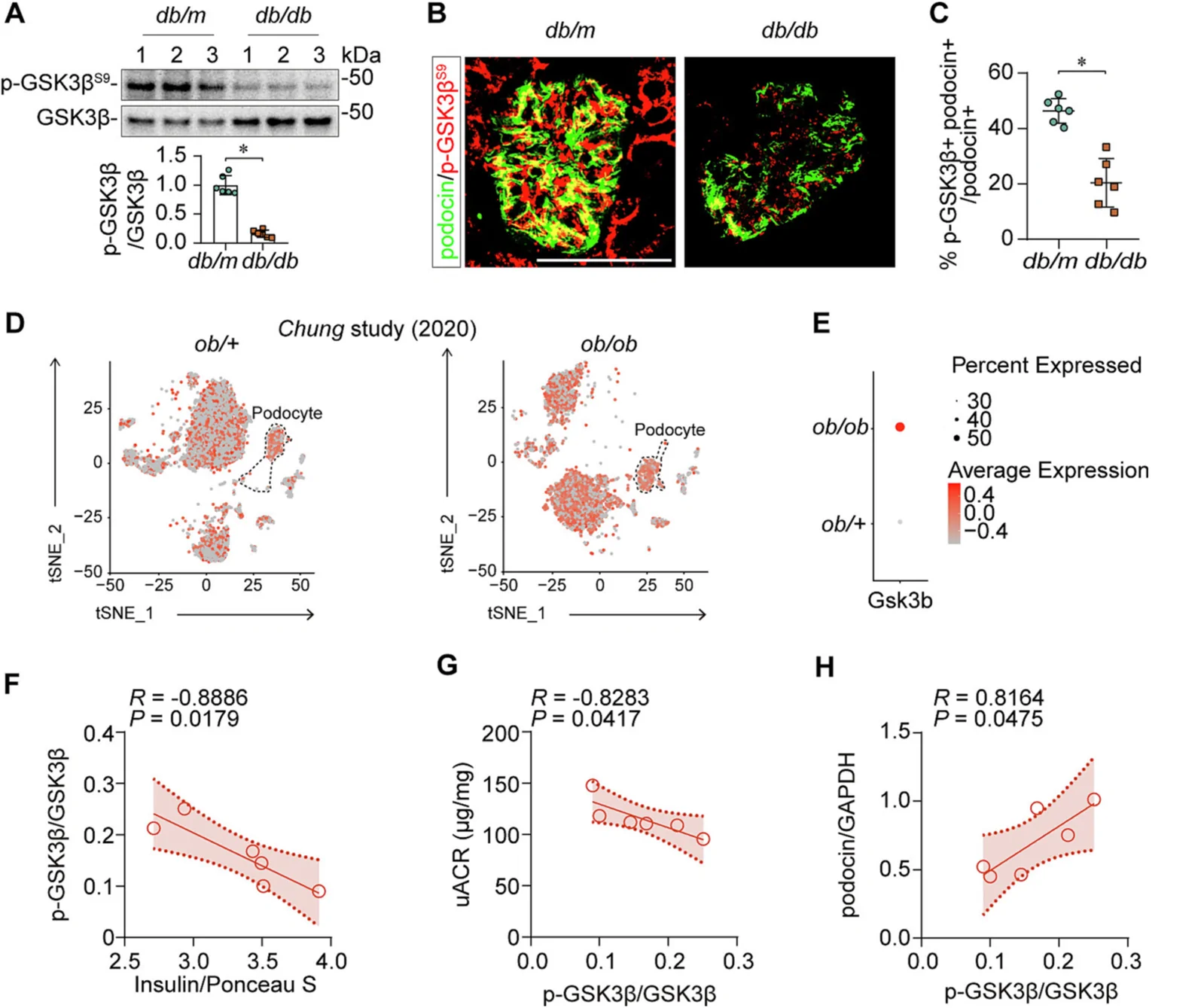
Glycogen synthase kinase (GSK) 3β overexpression and hyperactivity in glomerular podocytes correlate with hyperinsulinemia and albuminuria in prediabetic *db/db* mice. (A) Representative immunoblot analysis of glomeruli isolated from *db/m* and *db/db* mice at 6 weeks for indicated proteins. Densitometric analysis of the relative expression ratio of p-GSK3β^S9^/GSK3β based on immunoblot analysis. *P < 0.05 (n = 6). (B) Representative fluorescent micrographs of dual-color fluorescence staining for podocin (green) and p-GSK3β^S9^ (red) in frozen murine kidney sections (Scale bar, 50 μm). (C) The percentage of podocin co-localized with p-GSK3β^S9^ staining in glomeruli as measured by ImageJ. *P < 0.05 (n = 6). (D) t-distributed stochastic neighbor embedding (t-SNE) from single-cell RNA sequencing (scRNAseq) data of glomerular cells from *ob/ob* mice and control *ob/*+ mice at 12 weeks old. The distribution and relative expression of GSK3β in kidneys from *ob/*+ mice and ob/ob mice are shown. (E) Feature plots showing expression of GSK3β in podocytes across dataset in *ob/*+ mice and *ob/ob* mice at 12 weeks. (F) Linear regression analysis reveals significant correlations between the relative levels of insulin from Fig. 1B and the relative p-GSK3β^S9^/GSK3β ratios based on densitometric analysis of immunoblots in Fig. 3A (n = 6). Spearman's correlation coefficient (*R*) and *P* value are shown. (G) Linear regression analysis reveals significant correlations between the relative p-GSK3β^S9^/GSK3β ratios in Fig. 3A and the levels of uACR from Fig. 1C (n = 6). Spearman's correlation coefficient (*R*) and *P* value are shown. (H) Linear regression analysis reveals significant correlations between the relative p-GSK3β^S9^/GSK3β ratios in Fig. 3A and the relative expression levels of podocin from Fig. 1F (n = 6). Spearman's correlation coefficient (*R*) and *P* value are shown.

**Fig. 4.**
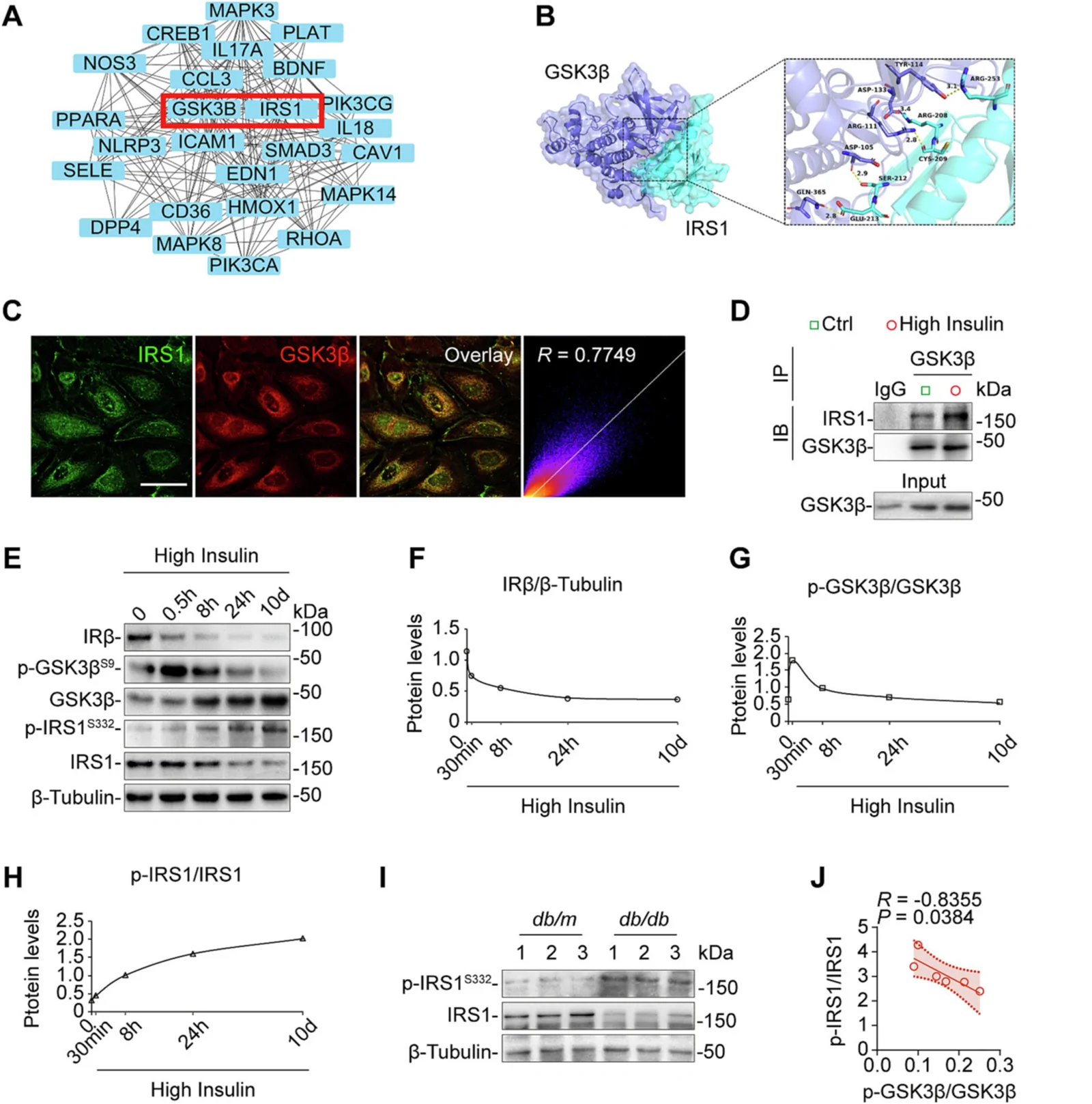
GSK3β colocalizes and physically interacts with insulin receptor substrate (IRS)1 in glomerular podocytes. (A) Visualization of the top 25 hub genes identified from the Protein-Protein Interaction network using Cytoscape. The red rectangle highlights the physical interaction between IRS1 and GSK3β. (B) Molecular docking analysis of GSK3β (purple) and IRS1 (green), with the putative binding sites zoomed within the dashed box. (C) Representative fluorescent micrographs of dual-color fluorescence staining for GSK3β (red) and IRS1 (green) in differentiated immortalized podocytes demonstrated that a discrete pool of cytoplasmic GSK3β co-localized with IRS1 (Scale bar, 50 μm). Frequency scatterplots of ImageJ co-localization analysis suggested a significant co-localization between GSK3β staining and IRS1 staining. Spearman's correlation coefficient (*R*) is shown. (D) Immortalized podocytes were treated with or without insulin (100 nM) for 10 days, in the presence of MG132 (10 μM) to prevent confounding proteasomal degradation. Cell lysates were subjected to immunoprecipitation (IP) with an anti-GSK3β antibody, followed by immunoblot (IB) analysis for IRS1 and GSK3β. Lysates from untreated cells immunoprecipitated with preimmune IgG served as negative controls. Equal amounts of cell lysates were used as input controls for immunoblot analysis. (E) Immortalized podocytes were treated with medium containing insulin (100 nM) for different times (0.5 h, 8 h, 24 h, 10 d) respectively. Representative immunoblot analysis of whole cell lysates for indicated proteins. β-Tubulin served as a loading control. (F) Densitometric analysis of relative expression of IRβ at different time points. (G) Densitometric analysis of the expression levels of p-GSK3β^S9^/GSK3β at different time points. (H) Densitometric analysis of the expression levels of p-IRS1^S332^/IRS1 at different time points. (I) Representative immunoblot analysis of whole lysates of isolated glomeruli from Fig. 3A for indicated proteins. β-Tubulin served as a loading control. (J) Linear regression analysis reveals significant correlations between the relative p-GSK3β^S9^/GSK3β ratios from Fig. 3A and the relative p-IRS1^S332^/IRS1 ratios based on densitometric analysis of immunoblots in Fig. 4I (*n* = 6). Spearman's correlation coefficient (*R*) and *P* value are shown.

**Fig. 5.**
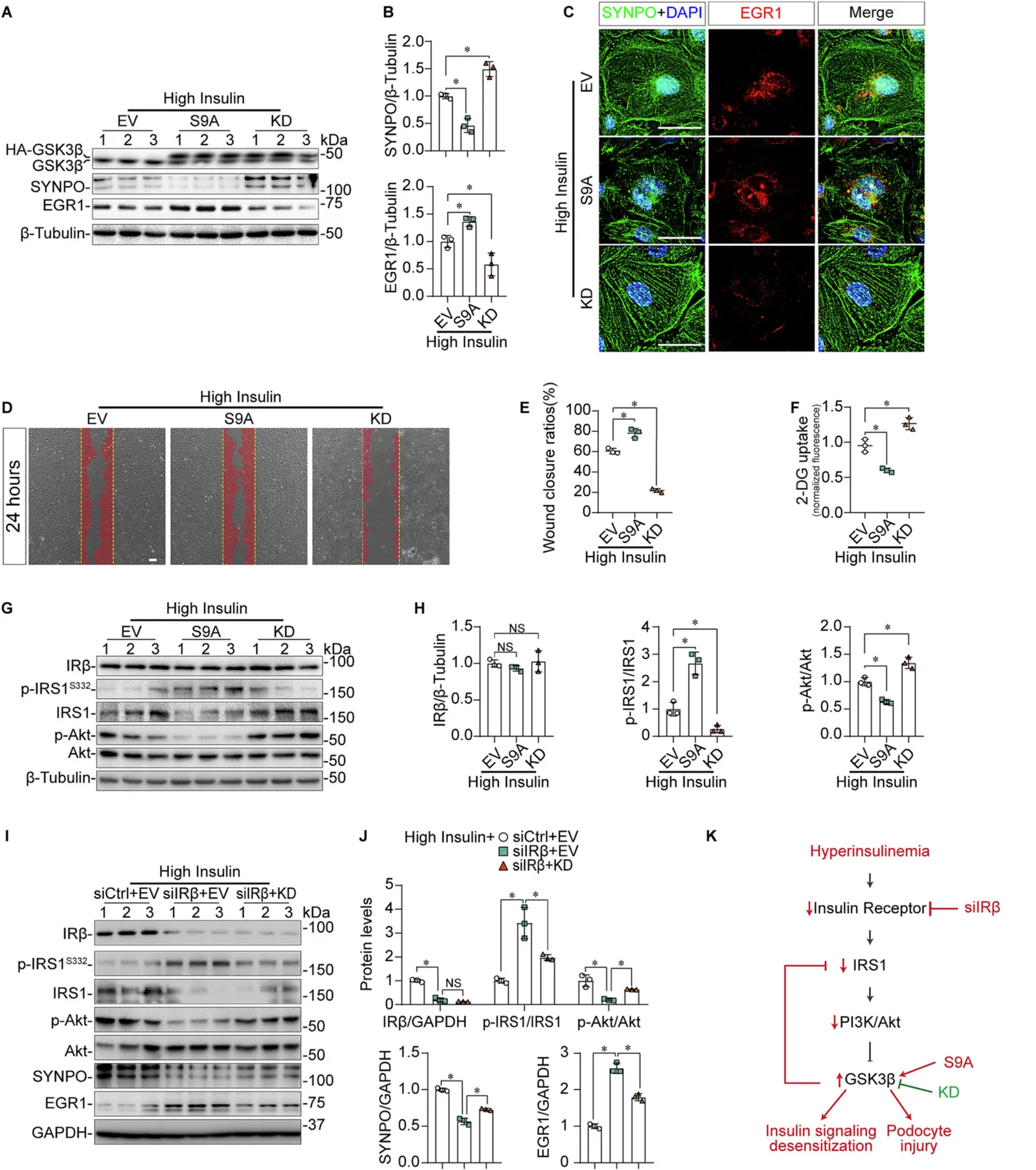
GSK3β regulates insulin signaling sensitivity and podocyte injury upon hyperinsulinemia insults. (A-H) Immortalized murine podocytes were transfected with plasmid vectors encoding an empty vector (EV) or HA-conjugated kinase-dead mutant of GSK3β (KD) or constitutively active mutant of GSK3β (S9A), and cultured in medium containing a high dose of insulin (100 nM) for 10 days. (A) Cell lysates from cultured podocytes were processed for immunoblot analysis for indicated proteins. β-Tubulin served as a loading control. (B) Densitometric analysis of the expression levels of the indicated proteins, presented as relative levels normalized to β-Tubulin based on immunoblot analysis. *P < 0.05 (*n* = 3). (C) Representative micrographs of immunofluorescence staining for synaptopodin (SYNPO, green) and early growth response 1 (EGR1, red) with 4′,6-diamidino-2-phenylindole (DAPI) counterstaining for nuclei (Scale bar, 50 μm). (D) Confluent monolayers of podocytes were scraped with a 10 μL pipette 30 min after different treatments. Representative micrographs taken at 24 h were shown. The cell migration areas were marked as red (Scale bar, 50 μm). (E) Computerized morphometric analysis of wounding areas following the indicated treatments. Data were expressed as the ratios of wound closure. *P < 0.05 (*n* = 3). (F) Quantitation of 2-DG uptake in cultured podocytes after indicated treatment. *P < 0.05 (n = 3). (G) Cell lysates from cultured podocytes were processed for immunoblot analysis for indicated proteins. β-Tubulin served as a loading control. (H) Densitometric analysis of the relative expression levels of p-IRS1^S332^/IRS1 and p-Akt/Akt. Expression of IRβ was presented as relative levels normalized to β-Tubulin based on immunoblot analysis. *P < 0.05 (n = 3). (I) Immortalized murine podocytes were subjected to co-transfection with siRNA against IRβ (siIRβ) or control siRNA (siCtrl) and plasmid vectors encoding either EV or HA-conjugated KD of GSK3β, followed by exposure to high dose of insulin (100 nM) for 10 days. Cell lysates were processed for immunoblot analysis for indicated proteins. GAPDH served as a loading control. (J) Densitometric analysis of the relative expression levels of p-IRS1^S332^/IRS1 and p-Akt/Akt. Expression of IRβ was presented as relative levels normalized to GAPDH based on immunoblot analysis. *P < 0.05 (*n* = 3). (K) Schematic illustration depicting the working model of a self-perpetuating signaling cycle underlying insulin desensitization and podocyte injury in the state of hyperinsulinemia, highlighting the central role of the GSK3β-IRS1 feedforward pathogenic loop driven by the hyperactive GSK3β, which promotes the inhibitory phosphorylation of IRS1^S332^. The deleterious effect of hyperinsulinemia or prolonged high insulin exposure could be mimicked by IRβ silencing-mediated insulin receptor depletion, exacerbated by expression of the constitutively active mutant of GSK3β (S9A), and attenuated by expression of kinase-dead GSK3β (KD).

**Fig. 6.**
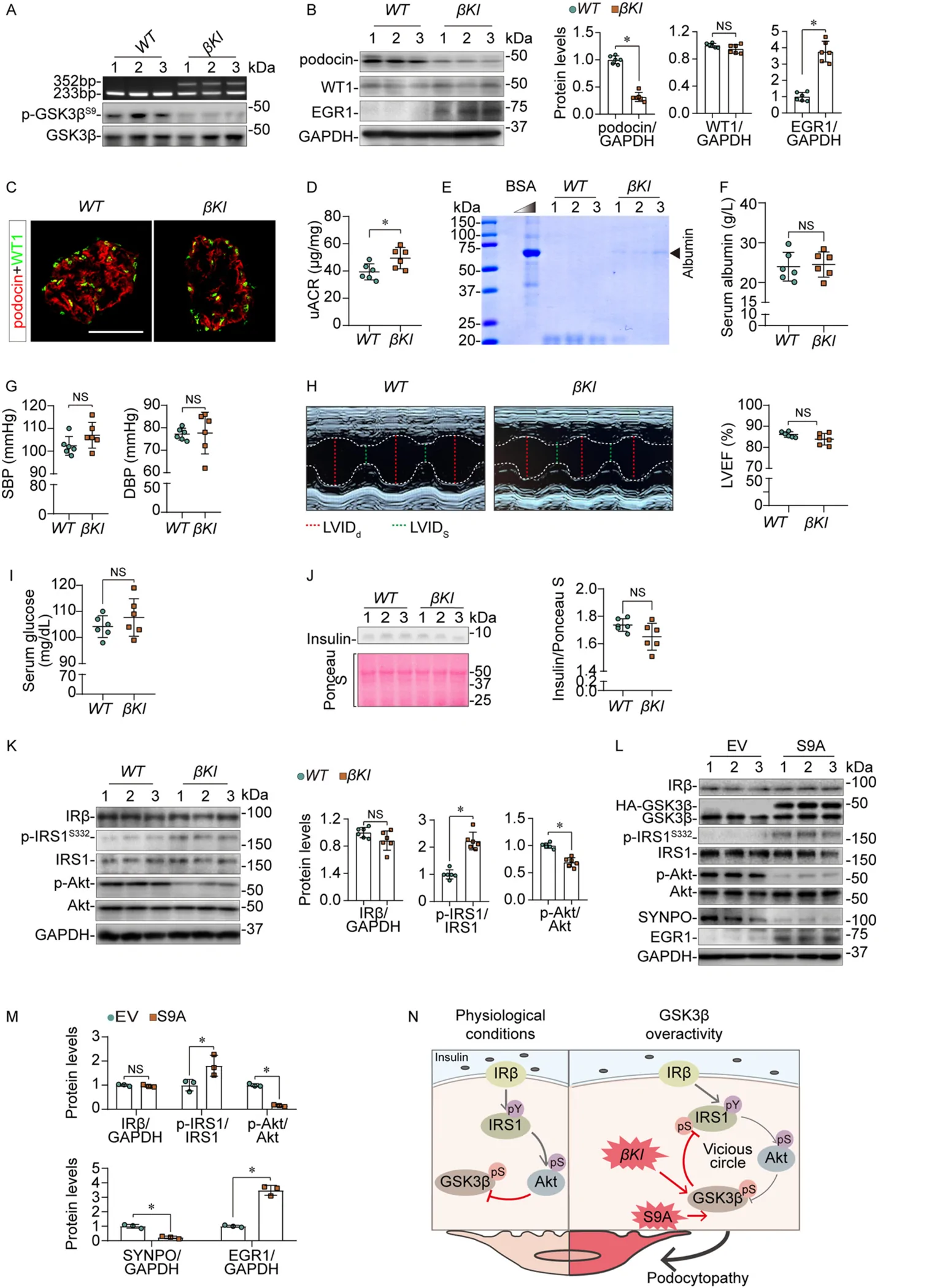
Constitutively active *GSK3β*^*S9A*^ *knock-in (βKI)* mice develops podocyte injury, insulin resistance and albuminuria in the absence of hyperinsulinemia or hyperglycemia. (A) Representative agarose gel electrophoresis showing genotyping of indicated mice. Representative immunoblot analysis of whole lysates of isolated glomeruli for indicated proteins. (B) Representative immunoblot analysis of whole lysates of isolated glomeruli for indicated proteins. GAPDH served as a loading control. Densitometric analysis of the expression levels of the indicated proteins, presented as relative levels normalized to GAPDH based on immunoblot analysis. *P < 0.05; NS, non-significance (*n* = 6). (C) Representative fluorescent micrographs of dual-color fluorescence staining for podocin (red) and WT1 (green) in frozen murine kidney sections (Scale bar, 50 μm). (D) Urinary albumin excretion as quantified by the uACR in *WT* and *βKI* mice. *P < 0.05 (n = 6). (E) Equal aliquots of spot urine were subjected to SDS-PAGE followed by Coomassie Brilliant Blue staining. Bovine serum albumin (BSA, 0.5 μg) served as standard. (F) Serum albumin levels in *WT* and *βKI* mice were tested. NS, non-significance (n = 6). (G) Systolic blood pressure (SBP) and diastolic blood pressure (DBP) levels in *WT* and *βKI* mice were tested. NS, non-significance (*n* = 6). (H) Representative M-mode echocardiographic imaging of the heart and the percentage of ejection fraction (EF) in *WT* and *βKI* mice. NS, non-significance (n = 6). (I) Serum glucose levels in *WT* and *βKI* mice were tested. NS, non-significance (n = 6). (J) Representative immunoblots analysis of serum insulin in *WT* and *βKI* mice. Ponceau S-stained blots served as a loading control. Densitometric analysis of the expression levels of insulin protein, presented as relative levels normalized to Ponceau S-stained blots based on immunoblot analysis. NS, non-significance (*n* = 6). (K) Representative immunoblot analysis of whole lysates of isolated glomeruli for indicated proteins. GAPDH served as a loading control. Densitometric analysis of the relative expression levels of p-IRS1^S332^/IRS1 and p-Akt/Akt. Expression of IRβ was presented as relative levels normalized to GAPDH based on immunoblot analysis. *P < 0.05; NS, non-significance (n = 6). (L) Immortalized murine podocytes were transfected with plasmid vectors encoding EV or HA-conjugated S9A mutant of GSK3β for 48 h. Cell lysates from cultured podocytes were processed for immunoblot analysis for indicated proteins. GAPDH served as a loading control. (M) Densitometric analysis of the expression levels of the indicated proteins, presented as relative levels normalized to GAPDH based on immunoblot analysis. * *P* < 0.05; NS, non-significance (*n* = 3). (N) Scheme depicting the proposed model for the key role of hyperactive GSK3β in insulin resistance and podocyte injury, highlighting the GSK3β-IRS1 self-reinforcing feedforward signaling cycle underlying insulin desensitization. Notably, even in the absence of hyperinsulinemia, *GSK3β*^*S9A*^ *KI* mice or podocytes expressing the S9A mutant recapitulate the impaired insulin signaling and podocyte injury observed in hyperinsulinemic mice or podocytes exposed prolonged high insulin.

**Fig. 7.**
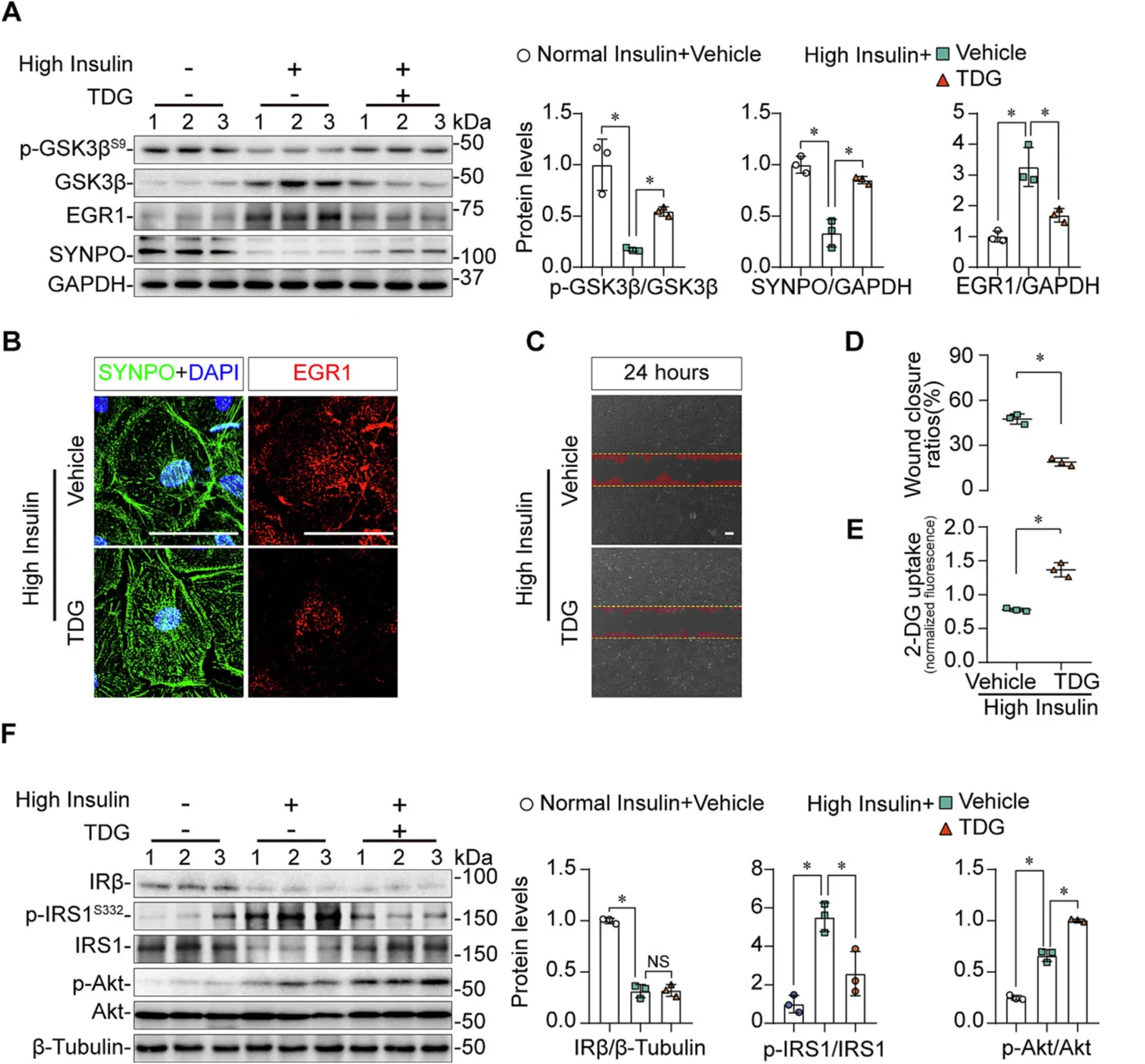
Tideglusib, a selective GSK3β inhibitor, partially restores insulin sensitivity and attenuates podocyte injury upon high insulin insults. Differentiated immortalized murine podocytes were exposed to RPMI 1640 medium, or the medium containing high dose of insulin (100 nM) for 10 days prior to treatment with or without tideglusib (TDG, 5 μM) for 48 h. (A) Cell lysates from cultured podocytes were processed for immunoblot analysis for indicated proteins, GAPDH served as a loading control. Densitometric analysis of the relative expression levels of p-GSK3β^S9^/GSK3β. Expressions of EGR1 and SYNPO was presented as relative levels normalized to GAPDH based on immunoblot analysis. *P < 0.05 (n = 3). (B) Representative micrographs of immunofluorescence staining for EGR1 (Red) or SYNPO (green) with DAPI counterstaining (Scale bar, 50 μm). (C) Confluent monolayers of podocytes were scraped with a 10 μL pipette 30 min after different treatment. Representative micrographs taken at 24 h were shown. The cell migration areas were marked as red (Scale bar, 50 μm). (D) Computerized morphometric analysis of wounding areas following the indicated treatments. Data were expressed as the ratios of wound closure. *P < 0.05 (n = 3). (E) Quantitation of 2-DG uptake in cultured podocytes after indicated treatment. *P < 0.05 (n = 3). (F) Cell lysates from cultured podocytes were processed for immunoblot analysis for indicated proteins, β-Tubulin served as a loading control. Densitometric analysis of the relative expression levels of p-IRS1^S332^/IRS1 and p-Akt/Akt. Expression of IRβ was presented as relative levels normalized to β-Tubulin based on immunoblot analysis. *P < 0.05; NS, non-significance (n = 3).

**Fig. 8.**
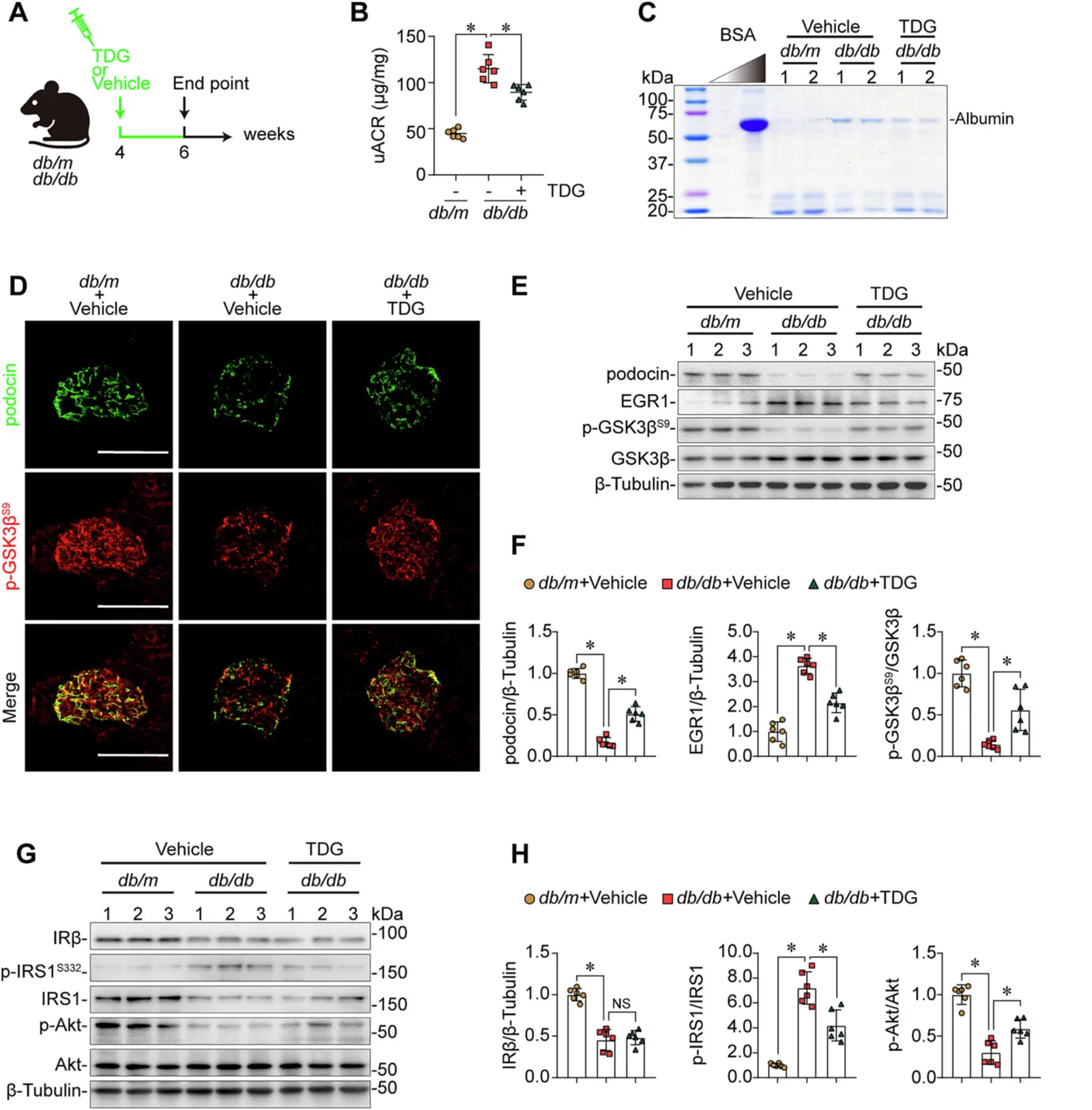
Tideglusib attenuates podocyte injury and insulin resistance in pre-diabetic *db/db* mice. (A) Schematic diagram illustrates the animal experimental design. *db/m* and *db/db* mice at 4 weeks old were randomized to receive TDG (5 mg/kg, QOD) or vehicle for 2 weeks. Mice were euthanized at 6 weeks of age. (B) Urinary albumin excretion was assessed by measuring uACR at the end point. *P < 0.05 (n = 6). (C) Spot urine collected at the end point was subjected to SDS-PAGE followed by Coomassie Brilliant Blue staining. Bovine serum albumin (BSA, 0.5 μg) served as a standard. (D) Representative fluorescent micrographs of dual-color fluorescence staining for podocin (green) and p-GSK3β^S9^ (red) in kidney cryosections (Scale bar, 50 μm). (E) Representative immunoblot analysis of isolated glomeruli for indicated proteins. β-Tubulin served as a loading control. (F) Densitometric analysis of the expression levels of the indicated proteins, presented as relative levels normalized to β-Tubulin based on immunoblot analysis. *P < 0.05 (n = 6). (G) Representative immunoblot analysis of isolated glomeruli for indicated proteins. β-Tubulin served as a loading control. (H) Densitometric analysis of the expression levels of the indicated proteins, presented as relative levels normalized to β-Tubulin based on immunoblot analysis. *P < 0.05; NS, non-significance (n = 6).

**Fig. 9.**
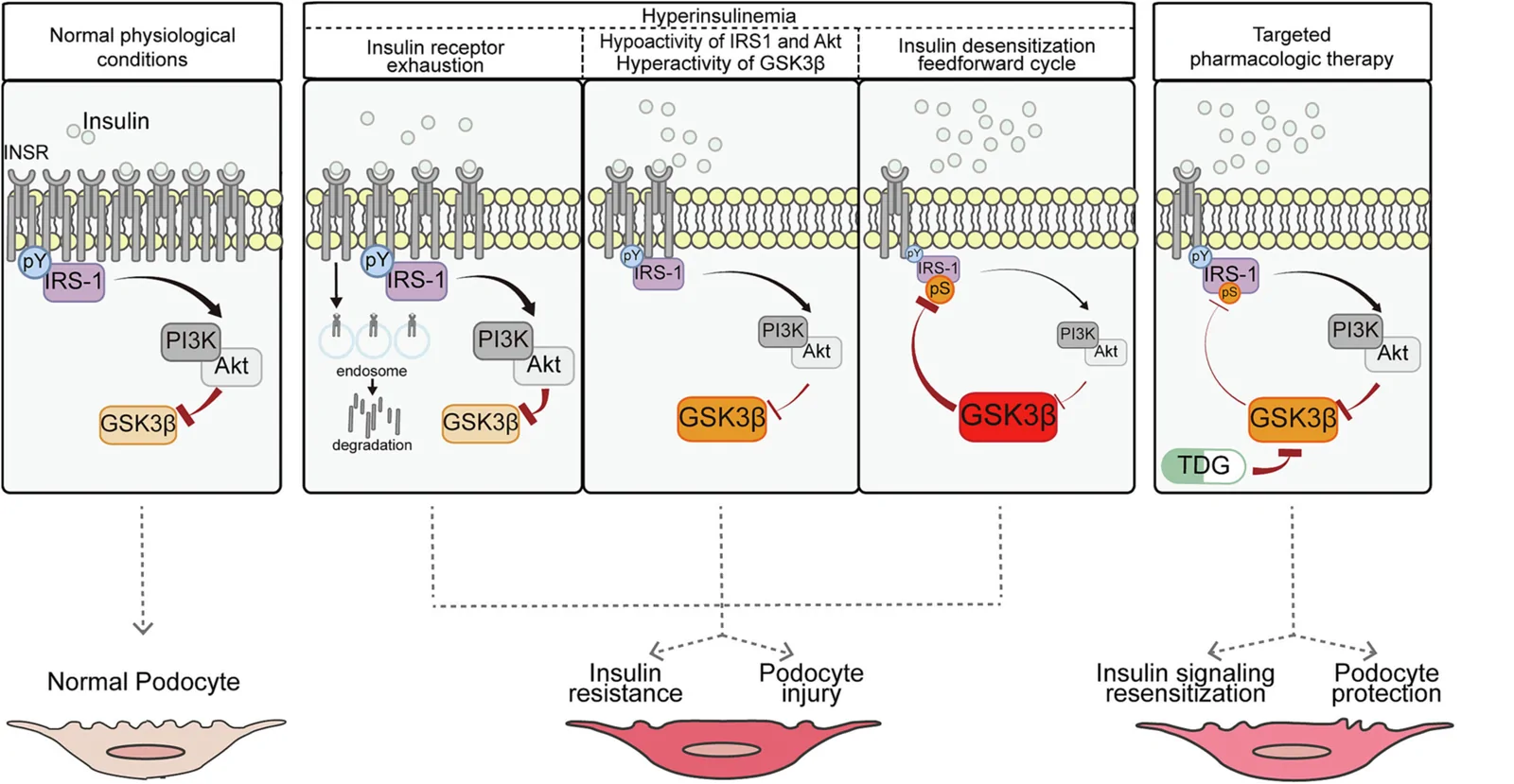
A schematic illustration depicting the self-perpetuating pathogenic signaling loop underlying hyperinsulinemia-induced podocyte injury and insulin resistance, highlighting the potential therapeutic benefit of targeting GSK3β. Under normal physiological conditions, insulin binding to IRβ facilitates the recruitment and activation of IRS1. This activation subsequently initiates a downstream signaling cascade involving phosphoinositide-3-kinase (PI3K) and Akt, ultimately leading to inhibitory phosphorylation of GSK3β in glomerular podocytes. In the state of hyperinsulinemia, chronic overstimulation of IRβ by prolonged insulin exposure results in insulin receptor exhaustion and desensitization of the downstream insulin signaling pathway. This desensitization weakens the inhibitory control of GSK3β, leading to its hyperactivity. As a cognate substrate of GSK3β, IRS1 undergoes increased inhibitory phosphorylation of IRS1^S332^ by the overactive GSK3β, culminating in IRS1 inhibition and repression of IRS1/PI3K/Akt signaling. The resulting enhancement of GSK3β activity establishes a self-perpetuating deleterious feedforward signaling loop that exacerbates insulin resistance and podocyte injury. Conversely, therapeutic targeting of GSK3β with the highly selective inhibitor tideglusib (TDG) intercepts the pathogenic loop, restores insulin sensitivity, and protects against hyperinsulinemia-induced podocyte injury.

## Appendix A. Supplementary data

Supplementary data to this article can be found online at https://doi.org/10.1016/j.metabol.2026.156571.
