# Supplementary material for: Hyperinsulinemia drives glomerular podocyte injury and albuminuria via a self-perpetuating GSK3β–IRS1 insulin desensitization circuit
Source: Metabolism. Author manuscript; Available in PMC 2026 Oct 28. (PMC13715427; doi:10.1016/j.metabol.2026.156571)
Supplement: Supplementary file 1 [file NIHMS2178782-supplement-1.pdf]

Supplemental Methods

2.1. Animal experiment

The mouse model of streptozotocin (STZ)-induced type 1 diabetic kidney disease had been generated in a previous study[1]. In brief, *C57BL/6* w*ild-type* male mice at 8 weeks old received daily intraperitoneal injection of low-dose STZ (55 mg/kg, Sigma Aldrich, St. Louis, MO, USA) for 5 days and were euthanized 8 weeks after STZ injection. Male mice treated with sodium citrate buffer served as controls. Archived specimens, including frozen homogenates of isolated glomeruli and kidney cryosections, were retrieved and examined.

Male mice were used in this study because females exhibit significant protection against metabolic disorder-related complications, such as hyperinsulinemia, diabetes and kidney disease[2]. For all animals, blood samples were collected at indicated time points and processed for measuring blood glucose levels.

2.2. Urine protein analyses

Spot urine was collected and underwent SDS-PAGE followed by Coomassie Brilliant Blue (Sigma-Aldrich) staining to determine the protein compositions. Urinary albumin and serum concentrations were analyzed using a mouse albumin enzyme-linked immunosorbent assay (ELISA) quantitation kit (Bethyl Laboratories Inc., Montgomery, TX, USA). Urine albumin concentrations were corrected for the urine creatinine concentrations assayed by using a creatinine assay kit (BioAssay System, Hayward, CA, USA).

2.3. Isolation of glomeruli

Glomerular isolation from mice was performed as previously described[3] with minor modifications. In brief, mice were euthanized by inhalation of 5% CO_2 and immediately perfused *via* left ventricular cannulation with ice-cold sterile phosphate-buffered saline (PBS) until the kidneys had blanched. The left renal pedicle was then ligated and the kidney resected for histological and other examinations. Subsequently, the right kidney was perfused *via* abdominal aorta cannulation with PBS containing magnetic iron oxide particles (Sigma-Aldrich)

using a syringe pump, and then resected for glomerular isolation. Renal cortices were minced into 1-mm³ pieces and digested in collagenase A at 37 °C for 30 minutes with gentle shaking. The digested tissue was gently pressed through 150-μm and 100-μm cell strainers (BD Falcon, Bedford, MA, USA), and glomeruli containing magnetic particles were collected using a magnetic particle concentrator. An aliquot of the glomerular isolate was visualized under a microscope to verify the cellular identity and purity to ensure that sample contained <5 tubular fragments per low-power field.

Then, enriched glomeruli were plated on collagen type I-coated Petri dishes at 37 °C in RPMI 1640 medium (Life Technologies, Grand Island, NY, USA) supplemented with 10% FBS, 1 mM sodium pyruvate (Sigma-Aldrich), 100 μg/mL streptomycin, and 100 U/mL penicillin (Life Technologies) in a humidified incubator with 5% CO_2. The glomeruli were then incubated in the presence or absence of high-dose insulin (100 nM, Thermo Fisher Scientific, Waltham, MA, USA)[4] for 30 minutes.

2.4. Bioinformatics analysis

Renal glomerular transcriptomic data from patients with diabetic nephropathy and healthy living donors in the Ju CKD Glom study[5] were analyzed using Nephroseq to evaluate glycogen synthase kinase 3β (GSK3β) expression profiles. Besides, glomerular transcriptomic data from *KK-A^{y} /TaJcl (KK-A^{y}*) mice with type 2 diabetes and control mice, generated by Sato et al.[6] (GSE261386), as well as those from mice with STZ-induced type 1 diabetes generated by Cao et al.[7] (GSE168676), were obtained from the Gene Expression Omnibus (GEO) database and analyzed to assess GSK3β expression profiles. In addition, human glomerular single-nucleus RNA sequencing (snRNA-seq) data from patients with early diabetic nephropathy and nondiabetic control subjects, generated by Wilson et al.[8], were accessed *via* the Kidney Interactive Transcriptomics (K.I.T.) (https://humphreyslab.com/SingleCell/) to assess the expression profiles of GSK3β and early growth response 1 (EGR1) in podocytes. Murine glomerular single-cell RNA sequencing (scRNA-seq)

data from 12-week-old BTBR.Cg-*Lep^ob/WiscJ* (*ob/ob*) and *ob/+* mice, generated by Chung et al.[9], were obtained from the Broad Institute Single Cell Portal (https://singlecell.broadinstitute.org/single_cell) under accession number SCP975, to analyze the expression profile of GSK3β in podocytes. Additionally, hyperinsulinemic podocyte injury–related genes were retrieved from the GeneCards database (https://www.genecards.org). The top 150 target genes were incorporated into the STRING database (https://string-db.org/) to construct a protein–protein interaction (PPI) network, and isolated nodes were removed. The top 25 hub genes, ranked by protein interaction score using the CytoHubba plugin, were identified with the Network Analyzer tool in Cytoscape software (version 3.10.2).

2.5. Molecular Docking

The X-ray crystal structure of GSK3β was obtained from the Protein Data Bank (https://www.rcsb.org/), and the predicted structure of insulin receptor substrate 1 (IRS1) was generated using AlphaFold. To ensure the accuracy of docking analysis, protein structures were preprocessed using AutoDockTools 1.5.7[10], with water molecules removed and polar hydrogens added. Protein–protein docking was performed using the GRAMM web server[11]. The resulting GSK3β–IRS1 complex was further optimized in AutoDockTools 1.5.7, and molecular interactions were analyzed in PyMOL. Functional residues at the binding interface were identified and classified according to interaction types. Multiple hydrogen bonds were identified between GSK3β and IRS1, including one between Arg111 of GSK3β and Cys209 of IRS1. The docking score of the GSK3β–IRS1 complex, obtained from the GRAMM server, was −454, consistent with a stable interaction.

2.6. Western immunoblot analysis and immunoprecipitation

Isolated renal glomeruli were homogenized, and immortalized mouse podocytes were lysed in RIPA buffer containing protease inhibitors (Roche Applied Science, Mannheim, Germany). Alternatively, isolated glomeruli

were processed for extracting nuclear fractions by using NE-PER kit (Thermo Fisher). Samples were analyzed by Western immunoblotting as previously described[12] using primary and secondary antibodies. Primary antibodies against Wilms' tumor 1 (WT1; Cat# sc-7385), podocin (Cat# sc-22298), synaptopodin (Cat# sc-515842), GAPDH (Cat# sc-32233), β-catenin (Cat# sc-7199) and histone H3 (Cat# sc-10809) were purchased from Santa Cruz Biotechnology (Dallas, TX, USA). Antibodies against EGR1 (Cat# 4154), IRβ (Cat# 3025), phospho-GSK3β (Ser9; Cat# 9336), GSK3β (Cat# 9315), IRS1 (Cat# 3407), phospho-IRS1 (Ser332/336; Cat# 2580), and β-tubulin (Cat# 2128) were purchased from Cell Signaling Technology (Danvers, MA, USA). Antibodies against phospho-Akt (Ser473; Cat# 66444-1-Ig) and Akt (Cat# 10176-2-AP) were obtained from Proteintech (Rosemont, IL, USA). Additionally, serum samples were incubated with the primary antibody against insulin (Cat# 3014, Cell Signaling Technology), with Ponceau-S staining serving as a loading control for serum insulin detection. Immunoprecipitation was performed as previously described[12], and the immunoprecipitates were subjected to immunoblot analysis for the indicated proteins.

2.7. Immunofluorescence staining

Cultured cells or tissue sections were fixed with 4% paraformaldehyde (Sigma-Aldrich), followed by permeabilization with 0.25% Triton X-100 in PBS buffer. Sections or cells were then incubated with primary antibodies against WT1 (Cat# sc-7385), podocin (Cat# sc-22298), and synaptopodin (Cat# sc-515842) obtained from Santa Cruz Biotechnology, or EGR1 (Cat# 4154), GSK3β (Cat# sc-377213), IRS1 (Cat# 3407) and phospho-GSK3β (Ser9; Cat# 9336) obtained from Cell Signaling Technology. This was followed by incubation with Alexa Fluor 488- or 594-conjugated secondary antibodies. After counterstaining with 4’,6-diamidino-2-phenylindole (DAPI; Abcam, Waltham, MA, USA), sections or cells were assessed in a blinded fashion by a single observer and imaged using a fluorescence microscope (EVOS XL Core Imaging System, Thermo Fisher) or a Leica TCS SP5 laser scanning confocal microscope.

2.8. Glucose uptake assay

Glucose uptake in glomeruli isolated from BKS.Cg-*Dock7*^{m} $^{+/+}$ *Lepr*^{db}/J (*db/m*) and *db/db* mice, or in cultured podocytes, was measured using a 2-deoxyglucose (2-DG) uptake assay kit (Abcam) according to the manufacturer's instructions. The fluorescence intensity derived from 2-DG oxidation was measured at excitation/emission wavelengths of 535/587 nm using a Cytation 5 imaging reader (Agilent Technologies Inc., Santa Clara, CA, USA).

2.9. Cell migration assay

Confluent monolayers of differentiated podocytes were scratched with a 10 μl pipette tip, subjected to the indicated treatments, and examined 24 h later using an EVOS FL microscope (Thermo Fisher Scientific). Phase-contrast images were captured at 0 h and 24 h post-scratch. The results were presented as the percentage of area closure, representing the relative reduction in wound area after 24 h, and analyzed using ImageJ (version 1.54; NIH, Bethesda, MD, USA). The percentage of cell-migration area was calculated as:

$$Wound\ Closure\ (\%) = \frac{A_{0h} - A_{24h}}{A_{0h}} \times 100$$

2.10. Blood pressure measurement

Blood pressure was measured using a non-invasive CODA tail-cuff plethysmography system (Kent Scientific, Torrington, CT, USA). Mice were placed on a heating platform for 10 min before measurement to enhance tail blood flow. To eliminate the influence of circadian rhythm, blood pressure was measured by the same operator at the same time of day. Each mouse was acclimated to the system for at least one week before measurement. A minimum of five measurements were taken until the blood pressure stabilized, and following stabilization, a minimum of eight additional measurements were taken per mouse and averaged for analysis.

2.11. Echocardiogram

Mice were anesthetized with isoflurane and underwent echocardiography in the supine position using an Acuson Sequoia C512 machine (Siemens) as previously described[13]. A 15 MHz linear transducer (15L8, Siemens) was used to acquire images in a shallow left-side position. Ejection fraction was obtained from the parasternal long-axis view by tracing the left ventricle in diastole and systole. The left ventricular ejection fraction (LVEF) was calculated using the Teichholz formula, based on left ventricular dimensions measured at end-diastole ($LVID_d$) and end-systole ($LVID_s$):

$$LVEF = ([100 - Y] \times 0.15) + Y; \; Y = (LVID_d^2 - LVID_s^2) \times 100/LVID_d^2.$$

References:

1. Fang, Y., et al., *The ketone body β-hydroxybutyrate mitigates the senescence response of glomerular podocytes to diabetic insults.* Kidney Int, 2021. **100**(5): p. 1037-1053.
2. Shepard, B.D., *Sex differences in diabetes and kidney disease: mechanisms and consequences.* Am J Physiol Renal Physiol, 2019. **317**(2): p. F456-f462.
3. Takemoto, M., et al., *A new method for large scale isolation of kidney glomeruli from mice.* Am J Pathol, 2002. **161**(3): p. 799-805.
4. Lay, A.C., et al., *Prolonged exposure of mouse and human podocytes to insulin induces insulin resistance through lysosomal and proteasomal degradation of the insulin receptor.* Diabetologia, 2017. **60**(11): p. 2299-2311.
5. Ju, W., et al., *Defining cell-type specificity at the transcriptional level in human disease.* Genome Res, 2013. **23**(11): p. 1862-73.
6. Sato, K., et al., *Glomerular Haematopoietic Prostaglandin D Synthase-Prostaglandin D2 Axis Contributes to the Periodontitis-Related Exacerbation of Diabetic Nephropathy in KK-A(y) Mice.* J Clin Periodontol, 2025. **52**(8): p. 1211-1220.
7. Cao, A., et al., *DACH1 protects podocytes from experimental diabetic injury and modulates PTIP-H3K4Me3 activity.* J Clin Invest, 2021. **131**(10).
8. Wilson, P.C., et al., *The single-cell transcriptomic landscape of early human diabetic nephropathy.* Proc Natl Acad Sci U S A, 2019. **116**(39): p. 19619-19625.
9. Chung, J.J., et al., *Single-Cell Transcriptome Profiling of the Kidney Glomerulus Identifies Key Cell Types and Reactions to Injury.* J Am Soc Nephrol, 2020. **31**(10): p. 2341-2354.
10. Morris, G.M., et al., *AutoDock4 and AutoDockTools4: Automated docking with selective receptor flexibility.* J Comput Chem, 2009. **30**(16): p. 2785-91.
11. Tovchigrechko, A. and I.A. Vakser, *GRAMM-X public web server for protein-protein docking.* Nucleic Acids Res, 2006. **34**(Web Server issue): p. W310-4.
12. Liu, Y., et al., *Endogenous hepatocyte growth factor ameliorates chronic renal injury by activating matrix degradation pathways.* Kidney Int, 2000. **58**(5): p. 2028-43.

13. Drummond, C.A., et al., *Na/K-ATPase signaling mediates miR-29b-3p regulation and cardiac fibrosis formation in mice with chronic kidney disease.* PLoS One, 2018. **13**(5): p. e0197688.

Supplmental Figures:

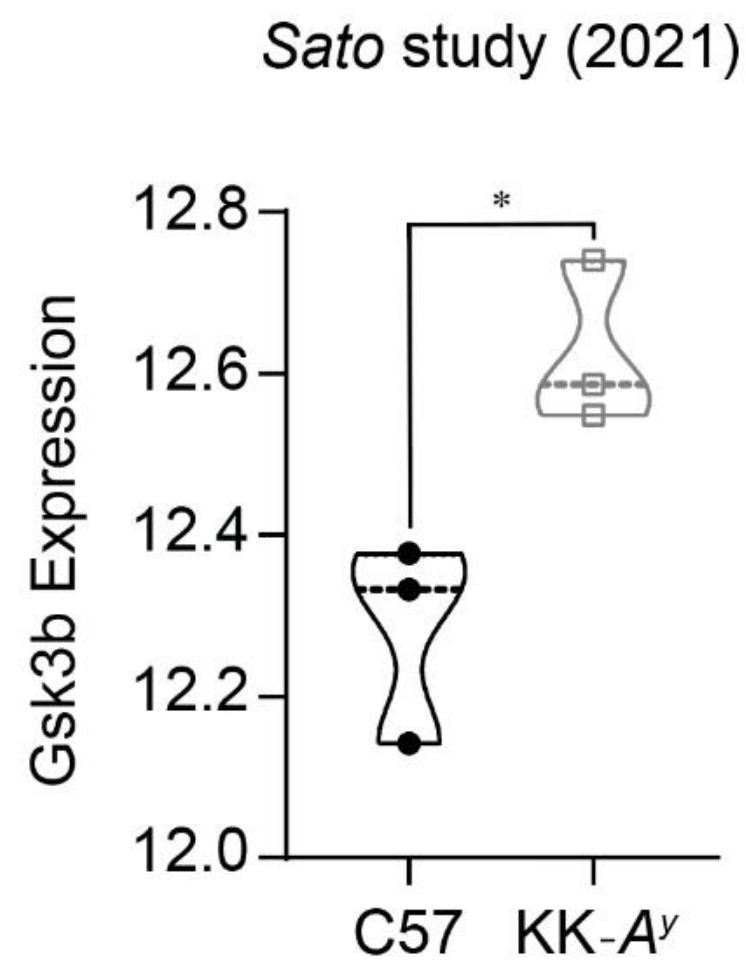

Supplemental Figure 1. Glomerular expression of GSK3β is increased in KK-*A*^{y} mice with type 2 diabetes. Gsk3b mRNA expression of Gsk3b for KK-*A*^{y} mice and C57 control mice were obtained from the Gene Expression Omnibus (GEO) database under accession number GSE261386 and were expressed as $\log_2$ median-centered intensity. *$P < 0.05$.

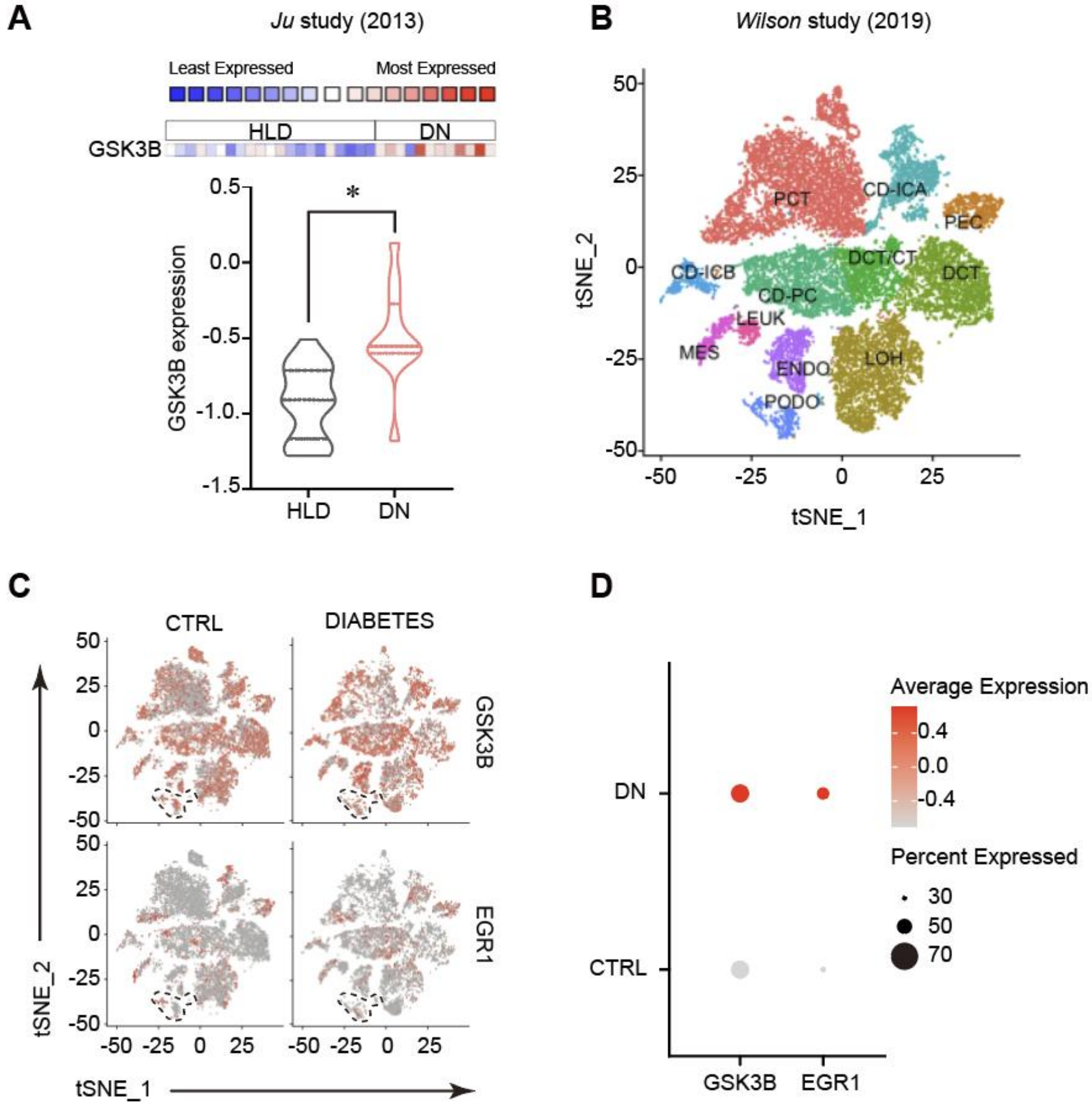

Supplemental Figure 2. GSK3β expression is increased in glomeruli and glomerular podocytes in patients with diabetic nephropathy.

(A) Renal glomerular transcriptomic data from patients with diabetic nephropathy (DN; n = 12) and healthy living donors (HLD; n = 21) in the Ju CKD Glom study were analyzed using Nephroseq to evaluate glycogen synthase kinase 3β (GSK3β) expression profiles. Data were derived from www.nephroseq.org on the basis of the Ju CKD Glom dataset and were expressed as $\log_2$ median-centered intensity. *$P < 0.05$. **(B-D)** Human glomerular single-nucleus RNA sequencing (snRNA-seq) data from patients with early diabetic nephropathy and nondiabetic control subjects, generated by Wilson et al., were accessed *via* the Kidney Interactive Transcriptomics (K.I.T.) (https://humphreyslab.com/SingleCell/) to assess the expression profiles of GSK3β and early growth response 1 (EGR1) in podocytes. **(B)** t-distributed stochastic neighbour embedding (t-SNE) plot of single-cell transcriptomes from kidneys of patients with early diabetic nephropathy (DN) and nondiabetic controls, obtained from the Kidney Interactive Transcriptomics (KIT) portal. Each dot represents a single cell and is colored according to the annotated cell-type clusters, including PCT (proximal convoluted tubule), CFH (complement factor H), LOH (loop of Henle), DCT (distal convoluted tubule), CT (connecting tubule), CD (collecting duct), PC (principal cell), IC (intercalated cell), PODO (podocyte), ENDO (endothelium), MES (mesangial cell) and LEUK (leukocyte). **(C)** Distribution and relative expression of GSK3B and EGR1 in diverse renal cells in kidneys from patients with early DN compared with non-diabetic control subjects. The cluster of podocytes is indicated by dashed lines. **(D)** Feature plots showing expression of GSK3B and EGR1 in podocytes from patients with early-stage Diabetes (DN) compared with non-diabetic control subjects (CTRL).

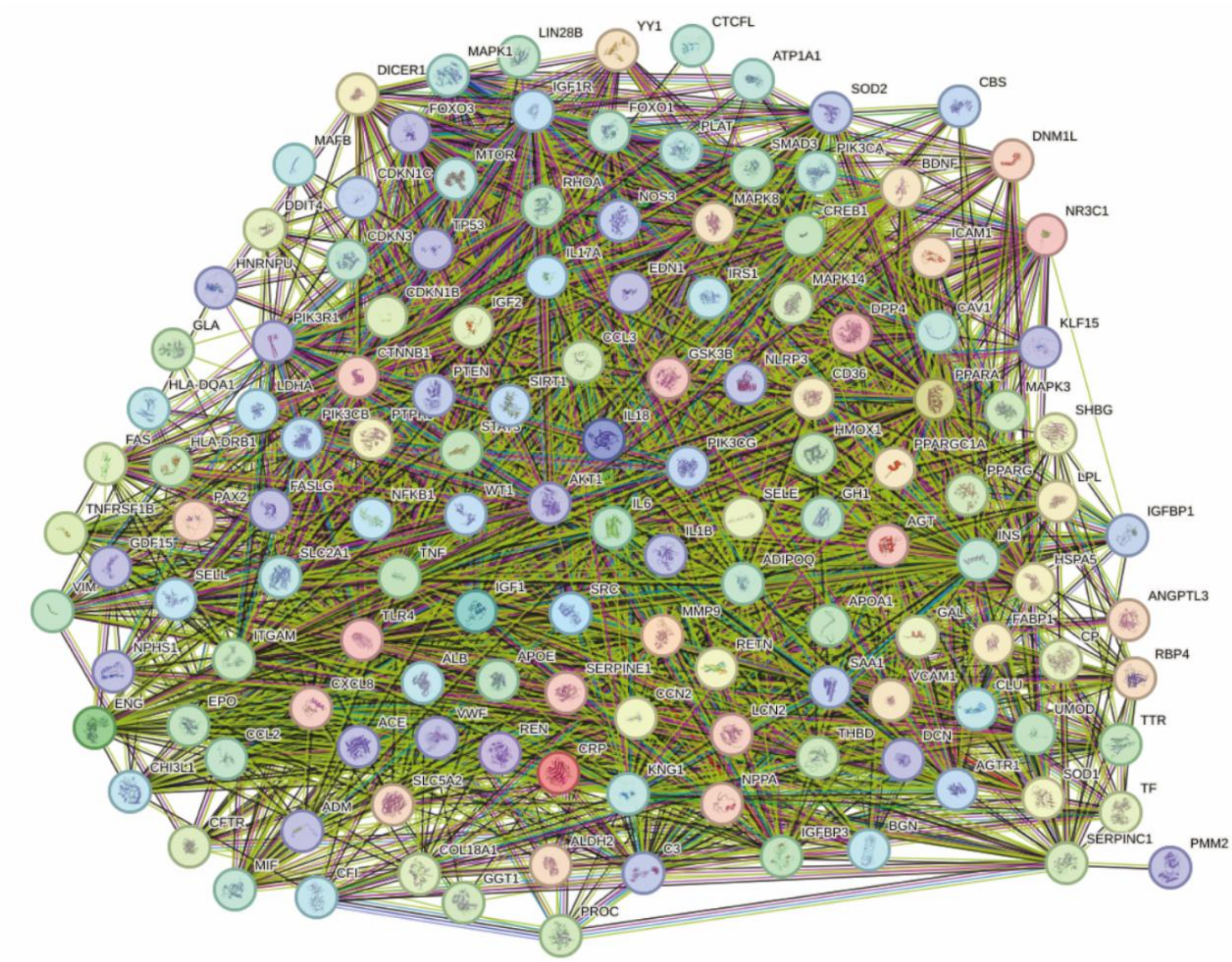

Supplemental Figure 3. GSK3β is predicted to be involved in hyperinsulinemia-induced podocyte injury. Hyperinsulinemic podocyte injury–related genes were retrieved from the GeneCards database. The top-ranked disease-associated genes were incorporated into the STRING database to construct a protein–protein interaction (PPI) network, in which GSK3β emerged as a potential central hub gene with a high interaction score.

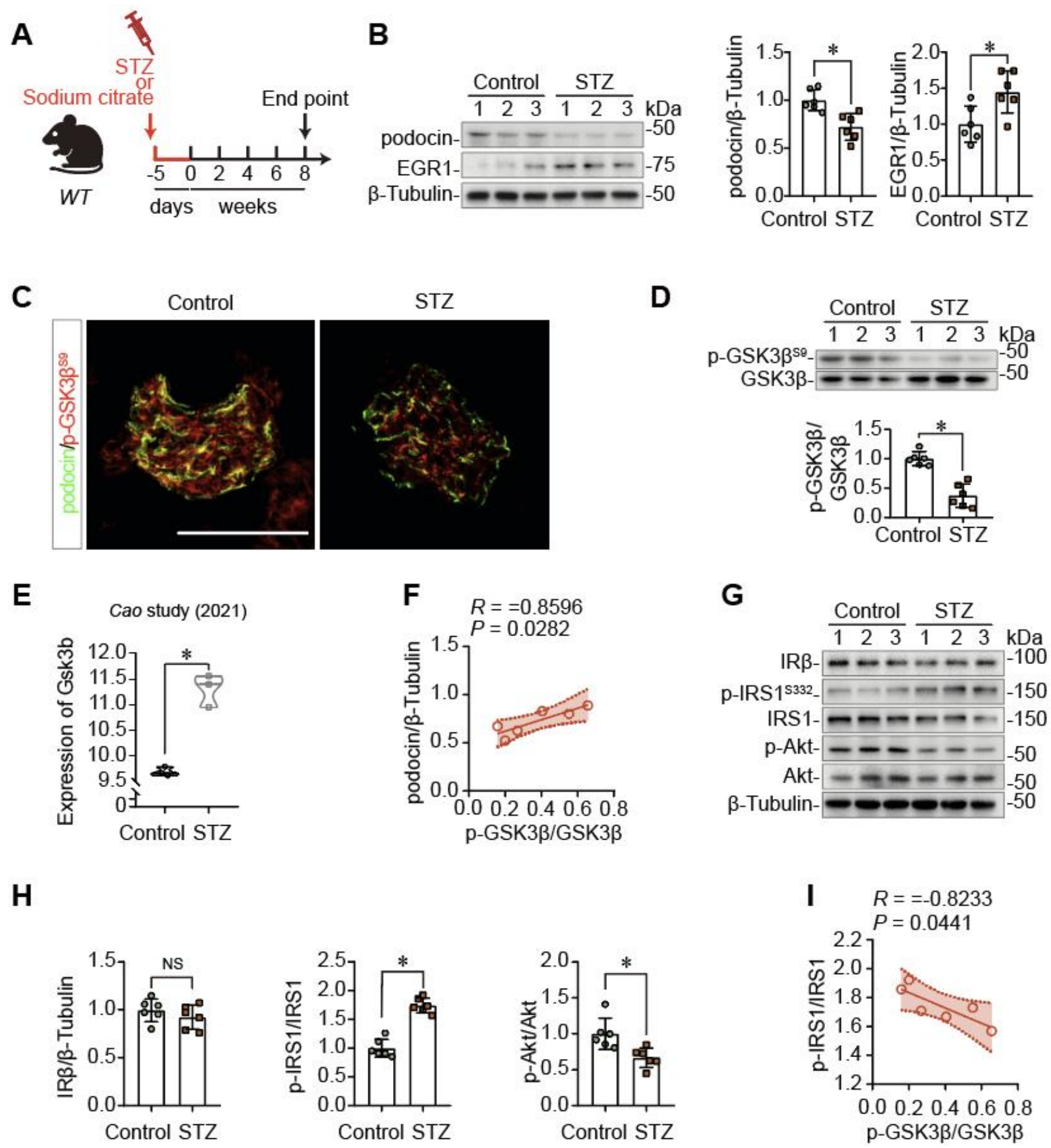

Supplemental Figure 4. Podocyte GSK3β hyperactivity also occurs in mice with STZ-induced type 1 diabetic kidney disease and contributes to impaired insulin signaling.

(A) Schematic diagram illustrates the animal experimental design. *Wild-type* (*WT*) mice were randomized to receive daily intraperitoneal injection of streptozotocin (STZ, 55 mg/kg/day) or an equal amount of sodium citrate as control for 5 consecutive days. Mice were euthanized 8 weeks after STZ injection. **(B)** Representative immunoblot analysis of isolated glomeruli for indicated proteins. Densitometric analysis of the expression levels of the indicated proteins, presented as relative levels normalized to β-Tubulin based on immunoblot analysis. $^{*}P < 0.05$ (n = 6). **(C)** Representative fluorescent micrographs of dual-color fluorescence staining for podocin (green) and p-GSK3β^{S9} (red) in frozen murine kidney sections (Scale bar, 50 µm). **(D)** Representative immunoblot analysis of isolated glomeruli for indicated proteins. Densitometric analysis of the relative expression ratio of p-GSK3β^{S9}/GSK3β based on immunoblot analysis. $^{*}P < 0.05$ (n = 6). **(E)** Glomerular transcriptomic data from mice treated with or without STZ, generated by Cao et al., were obtained from the publicly available GEO dataset (GSE168676) and analyzed to assess GSK3β expression profiles. Data were presented as $\log_2$ median-centered intensity. **(F)** Linear regression analysis reveals significant correlations between the relative ratios of glomerular p-GSK3β^{S9}/GSK3β in **Supplemental Figure 4D** and the relative expression levels of podocin from **Supplemental Figure 4B** (n = 6). Spearman's correlation coefficient (*R*) and *P* value are shown. **(G)** Representative immunoblot analysis of isolated glomeruli for indicated proteins. **(H)** Densitometric analysis of the expression levels of the indicated proteins, presented as relative levels normalized to β-Tubulin based on immunoblot analysis. $^{*}P < 0.05$; NS, non-significance (n = 6). **(I)** Linear regression analysis reveals significant correlations between the relative ratios of glomerular p-GSK3β^{S9}/GSK3β in **Supplemental Figure 4C** and the relative ratios of glomerular p-IRS1^{S332}/IRS1 based on densitometric analysis

of immunoblots in **Supplemental Figure 4H** (n = 6). Spearman's correlation coefficient (*R*) and *P* value are shown.

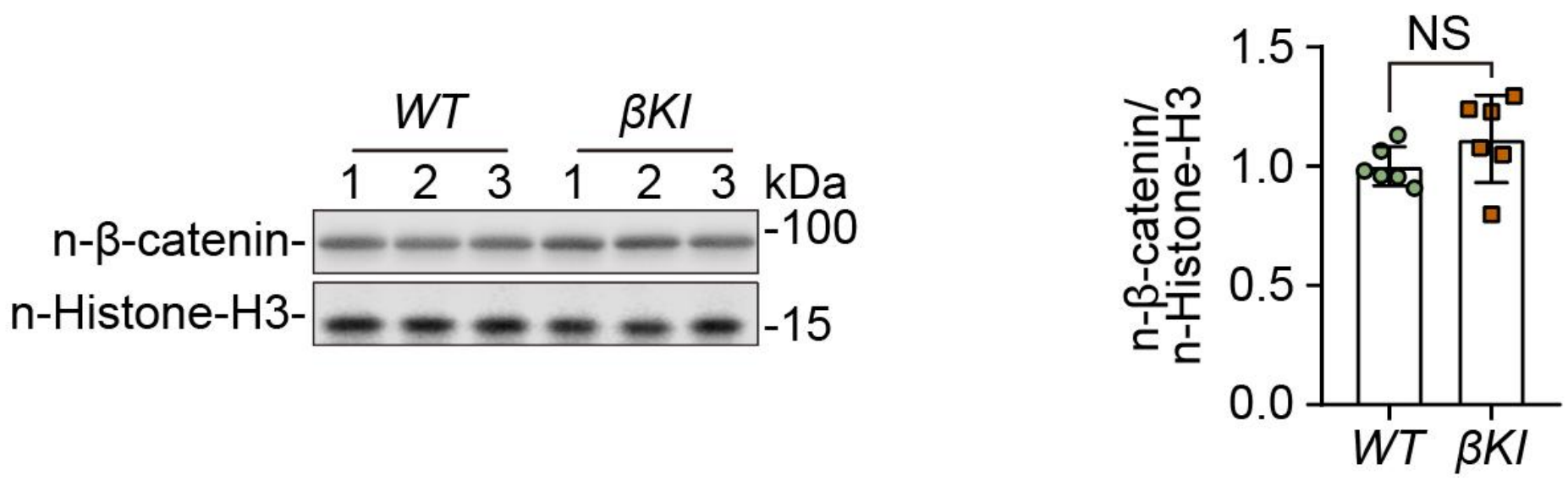

Supplemental Figure 5. GSK3β^{S9A} knock-in barely alters glomerular β-catenin activity in mice. Representative immunoblot analysis of nuclear fractions of glomeruli isolated from *WT* and *βKI* mice for nuclear β-catenin (n-β-catenin) as well as histone H3 (n-Histone H3), which served as a loading control. Densitometric analysis of the relative expression levels of n-β-catenin, presented as relative levels normalized to n-Histone H3 based on immunoblot analysis. NS, non-significance (n = 6).
